# Microbial co-occurrence complicates associations of gut microbiome with US immigration, dietary intake and obesity

**DOI:** 10.1186/s13059-021-02559-w

**Published:** 2021-12-10

**Authors:** Zheng Wang, Mykhaylo Usyk, Yoshiki Vázquez-Baeza, Guo-Chong Chen, Carmen R. Isasi, Jessica S. Williams-Nguyen, Simin Hua, Daniel McDonald, Bharat Thyagarajan, Martha L. Daviglus, Jianwen Cai, Kari E. North, Tao Wang, Rob Knight, Robert D. Burk, Robert C. Kaplan, Qibin Qi

**Affiliations:** 1 Department of Epidemiology and Population Health, Albert Einstein College of Medicine, 1300 Morris Park Avenue, Bronx, NY 10461 USA; 2 Departments of Pediatrics, Albert Einstein College of Medicine, Bronx, New York, USA; 3 Center for Microbiome Innovation, University of California, San Diego, La Jolla, CA USA; 4 Jacobs School of Engineering, University of California, San Diego, La Jolla, CA USA; 5 Public Health Sciences Division, Fred Hutchinson Cancer Research Center, Seattle, WA USA; 6 Department of Pediatrics, University of California, San Diego, La Jolla, CA USA; 7 University of Minnesota Medical Center, Minneapolis, MN USA; 8 University of Illinois at Chicago, Chicago, IL USA; 9 University of North Carolina at Chapel Hill, Chapel Hill, NC USA; 10 Department of Bioengineering, University of California, San Diego, La Jolla, CA USA; 11 Department of Computer Science and Engineering, University of California, San Diego, La Jolla, CA USA; 12 Department of Obstetrics & Gynecology and Women’s Health, Albert Einstein College of Medicine, Bronx, NY USA; 13 Department of Microbiology & Immunology, Albert Einstein College of Medicine, Bronx, NY USA; 14 Department of Nutrition, Harvard T.H. Chan School of Public Health, Boston, MA USA

**Keywords:** Microbiome, Metagenomics, Hispanic population, Obesity

## Abstract

**Background:**

Obesity and related comorbidities are major health concerns among many US immigrant populations. Emerging evidence suggests a potential involvement of the gut microbiome. Here, we evaluated gut microbiome features and their associations with immigration, dietary intake, and obesity in 2640 individuals from a population-based study of US Hispanics/Latinos.

**Results:**

The fecal shotgun metagenomics data indicate that greater US exposure is associated with reduced ɑ-diversity, reduced functions of fiber degradation, and alterations in individual taxa, potentially related to a westernized diet. However, a majority of gut bacterial genera show paradoxical associations, being reduced with US exposure and increased with fiber intake, but increased with obesity. The observed paradoxical associations are not explained by host characteristics or variation in bacterial species but might be related to potential microbial co-occurrence, as seen by positive correlations among *Roseburia*, *Prevotella*, *Dorea*, and *Coprococcus*. In the conditional analysis with mutual adjustment, including all genera associated with both obesity and US exposure in the same model, the positive associations of *Roseburia* and *Prevotella* with obesity did not persist, suggesting that their positive associations with obesity might be due to their co-occurrence and correlations with obesity-related taxa, such as *Dorea* and *Coprococcus*.

**Conclusions:**

Among US Hispanics/Latinos, US exposure is associated with unfavorable gut microbiome profiles for obesity risk, potentially related to westernized diet during acculturation. Microbial co-occurrence could be an important factor to consider in future studies relating individual gut microbiome taxa to environmental factors and host health and disease.

**Supplementary Information:**

The online version contains supplementary material available at 10.1186/s13059-021-02559-w.

## Background

Evidence from animal studies suggests that the gut microbiome may play a causal role in the development of obesity [1, 2], while the relationship between gut microbiome and obesity remains unclear in humans [3, 4]. Recent systematic reviews found mixed results on gut microbiome features associated with obesity across human studies [3, 4]. The inconsistency in human studies may be due to small sample sizes (the majority of previous studies had *N* < 100) and/or large interpersonal variation in gut microbiome related to many host factors (e.g., race/ethnicity [5], geography [6], diet/lifestyle [7, 8]). On the other side, microbes do not live in isolation but develop a range of relationships in the communities, including mutualism, commensalism, synergism, competition parasitism, predation, antagosim, and amensalism [9], and the complex microbial interaction might also influence the relationship of gut microbiome with obesity and other conditions.

Emerging evidence suggests that immigration is associated with gut microbiome alterations, which might be related to changes in diet and other factors during the acculturation process [10, 11]. Notably, microbiome-related conditions such as obesity and its comorbidities are major health concerns among US immigrants from lower-to-middle income countries [12]. A recent study in immigrants from Thailand to the USA found that US immigration was associated with loss of gut microbiome diversity and reduced ratio of *Prevotella* to *Bacteroides*, and loss of gut microbiome diversity was associated with obesity [10]. These results suggested a potential involvement of gut microbiota alterations in immigration-related obesity [10]. However, not much is known about individual taxonomic signals and functional components related to obesity. Higher *Prevotella* relative abundance has been consistently reported in non-industrialized populations, which are more metabolically healthy (e.g., lower prevalence of obesity and diabetes compared to industrialized populations), and whose diets contain more dietary fiber [13–15]. Paradoxically, several studies have found positive associations of *Prevotella* with obesity [16, 17], insulin resistance [18], and inflammatory autoimmune diseases [19, 20]. The mechanisms underlying the unexpected association between gut *Prevotella* and human diseases are not fully understood [21–23].

This report focuses on the US Hispanic/Latino community, which represent a substantial fraction of the total immigrant population in the USA (https://www.pewresearch.org/hispanic). In US Hispanics/Latinos, the prevalence of obesity is particularly high, and the length of duration of US residence or US exposure at earlier ages is associated with higher prevalence of obesity as well as greater severity of obesity [24, 25]. Our prior work using 16S rRNA data in 1674 US Hispanics/Latinos showed that the ratio of *Prevotella* to *Bacteroides* is lower in US-born versus immigrant Hispanics/Latinos, potentially related to lower fiber intake [11]. However, this ratio was found to be higher in individuals with obesity compared to those with normal weight, which was paradoxical to the observation that US-born Hispanics/Latinos had a higher prevalence of obesity and lower fiber intake compare to immigrant Hispanics/Latinos [11]. Therefore, studies are needed to explore potential explanations for these paradoxical results. Particularly, microbial interactions have been commonly observed [26, 27], but have not been well-considered in the analyses of gut microbiome and human health and disease. Microbial co-occurrence network which infer ecological relationships based on taxonomic composition data obtained from high-throughput sequencing techniques [28], has been used to visualize the correlations between microbes in microbial communities and predict the potential microbial interactions [9].

To better understand the interrelationship among immigration, gut microbiome, and obesity in US Hispanics/Latinos, we extended our analyses using shotgun metagenomics sequencing on a larger group of individuals. Among 2640 US Hispanics/Latinos of diverse background (including Dominican, Cuban, Puerto Rican, Mexican, Central American, and South American), aged 23 to 83 years, from the Hispanic Community Health Study/Study of Latinos (HCHS/SOL ) [29, 30], we evaluated multiple gut microbiome features including community-level diversity, individual taxa, and functional potential, and examined associations of these features with immigration generation, duration of US residence, usual dietary intake, and obesity. The potential microbial co-occurrence relationship and its potential influences on the associations of gut microbiome with US immigration, dietary intake, and obesity were also examined.

## Results

### US exposure, obesity, and gut microbiome diversity

Among 2640 US Hispanics/Latinos (age ranged from 23 to 83 years), 368 (14%) were born in the mainland USA. Severity and prevalence of obesity were highest for those born in USA, as well as being positively associated with duration of US residence. Detailed study participant characteristics are shown in Additional file 1: Table S1.

Higher levels of obesity were associated with lower α-diversity Faith’s phylogenetic distance (PD) after multivariable adjustment in weighted linear regression (Additional file 1: Figure S1A). Consistently, α-diversity Faith’s PD index was lowest in those born in USA, and inversely associated with duration of US residence (Additional file 1: Figure S1A). Principal-coordinates analysis (PCoA) of weighted UniFrac distances indicated that both host obesity and US exposure (US born and duration of US residence) significantly co-varied with the β-diversity of gut microbiome and explained moderate proportion of variance (PERMANOVA analysis by adonis function from vegan package, R^2^ = 0.9% and 1.8% respectively, both P < 0.001) (Additional file 1: Figure S1B). These results are in line with previous findings in US immigrants from Thailand [10]. In addition, we also examined associations of other participant characteristics with β-diversity, and most variables were significantly associated with β-diversity, while all these R^2^ values were relatively small (R^2^ < 2.0%), including those for obesity and US exposure (Additional file 1: Figure S2). These results are consistent with those observed in our previous analyses using 16S data [11] as well as previous findings observed in large population-based studies in which multiple host factors showed significant associations with β-diversity but only explained a very small proportion of variation in β-diversity [6].

### US exposure, obesity, and gut bacterial genera

**Fig. 1:**
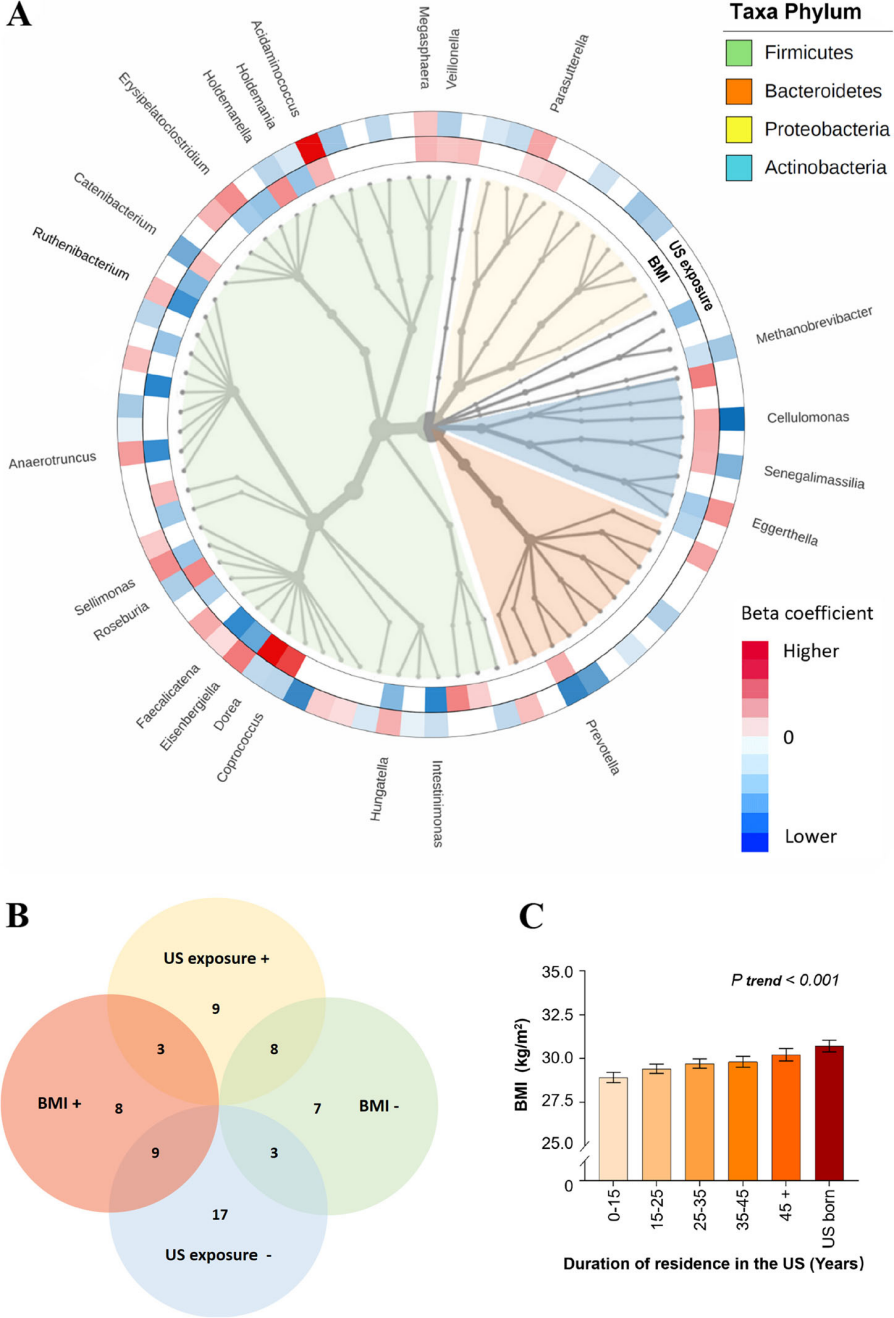
Associations of gut bacterial taxa with host obesity and US exposure. **A** Integrated tree of microbial communities associated with host obesity and US exposure. Taxa from inner to outer circle represent bacteria kingdom to genus level; 84 predominant genera (average relative abundance > 0.01%) were included. The branch widths reflect the relative abundance of each detected taxon. Red/blue colors of the rings depict significant positive/inverse associations and the gradient colors reflect the ranks of effect sizes estimated in linear regression models after adjustment for demographic, socioeconomic, behavioral, and clinical factors, while white color indicates non-significant associations. The inner ring shows 38 genera significantly associated with host body mass index (BMI) (FDR *P* < 0.05), and the outer ring shows 49 genera significantly associated with US exposure (defined by place of birth and duration of residence in the US) (FDR *P* < 0.05). A total of 23 genera were significantly associated with both BMI and US exposure and the genera names are indicated. **B** Venn diagram of gut bacterial genera associated with BMI and US exposure. The symbol “+”/“−” depict significant positive/inverse associations in linear regression models (FDR *P* < 0.05). **C** Associations between host obesity and US exposure. Data are adjusted mean (SE) of BMI across US exposure groups estimated in linear regression models after adjustment for demographic, socioeconomic, behavioral, and clinical factors

We then examined associations of individual gut microbial taxa at a genus level with obesity and US exposure after controlling for demographic, socioeconomic, behavioral, and clinical variables using weighted linear regression models. Of the 84 predominant gut bacterial genera included in this analysis (relative abundance ≥0.01%), 38 were significantly associated with BMI (FDR < 0.05; 26 under *Firmicutes* phylum, 6 under *Actinobacteria*, and 3 under *Proteobacteria*), and 49 genera were significantly associated with US exposure (FDR < 0.05; 33 under *Firmicutes* phylum, 6 under *Proteobacteria*, and 6 under *Bacteroidetes*) (Fig. 1A and Additional file 2: Table S2). Cross-classification of these two sets of results identified 23 bacterial genera that showed significant associations with both BMI and US exposure (Fig. 1A, B and Additional file 1: Table S3). Despite a strong positive association between BMI and US exposure (Fig. 1C), we found that a majority of genera (17 of 23) showed paradoxical associations with obesity and US exposure, including nine genera (e.g., *Roseburia*, *Prevotella*, and *Dorea*) associated with higher BMI but less US exposure, and the other eight genera (e.g., *Anaerotruncus* and *Eggerthella*) associated with lower BMI but greater US exposure. After further adjustment for Hispanic background, the associations of gut bacterial genera with obesity and US exposure did not change materially (Additional file 3: Table S4). In addition, in consideration of close correlations between US exposure and the covariates included in our regression models, we also compared associations of gut bacterial genera with US exposure and these covariates. Forty-nine genera were significantly associated with US exposure independently of these covariates, and their associations with these covariates are also shown in Additional file 4: Table S5.

To explore potential explanations for the observed paradoxical associations, we first examined these associations stratified by age, sex, geographic location, and Hispanic background as these factors may influence associations between gut microbiome features and human phenotypes. However, we did not find significant heterogeneity in associations of these bacterial genera with obesity (Additional file 1: Figure S3, and Additional file 5: Table S6) or US exposure (Additional file 3: Figure S4, and Additional file 6: Table S7) across strata. We also examined the associations of these bacterial genera with obesity and US exposure among the first-generation Hispanic/Latino immigrants according to the place of birth (i.e., Dominica, Cuba, Puerto Rica, Mexico, Central America, and South America), and did not find significant heterogeneity in these associations (Additional file 7: Table S8 and Additional file 8: Table S9). In addition, we performed sensitivity analyses by excluding individuals with antibiotic use during the past 6 months, and the results on the associations of gut bacteria genera with obesity and US exposure were similar to those of the primary analyses after adjustment for antibiotic use in the regression models. These results suggest that these host factors are less likely to explain the observed paradoxical associations.

We also examined a total of 88 species within these 23 genera but found little evidence suggesting that variation in species levels explain the observed paradoxical associations.

Results at the genus level were mostly driven by one or two major species under corresponding genera (Additional file 1: Table S10). For example, *Prevotella copri*, the most predominant species in *Prevotella* genus (mean relative abundance: 11%, accounting for 88.3% of *Prevotella* species), was associated with higher BMI but less US exposure.

**Fig. 2:**
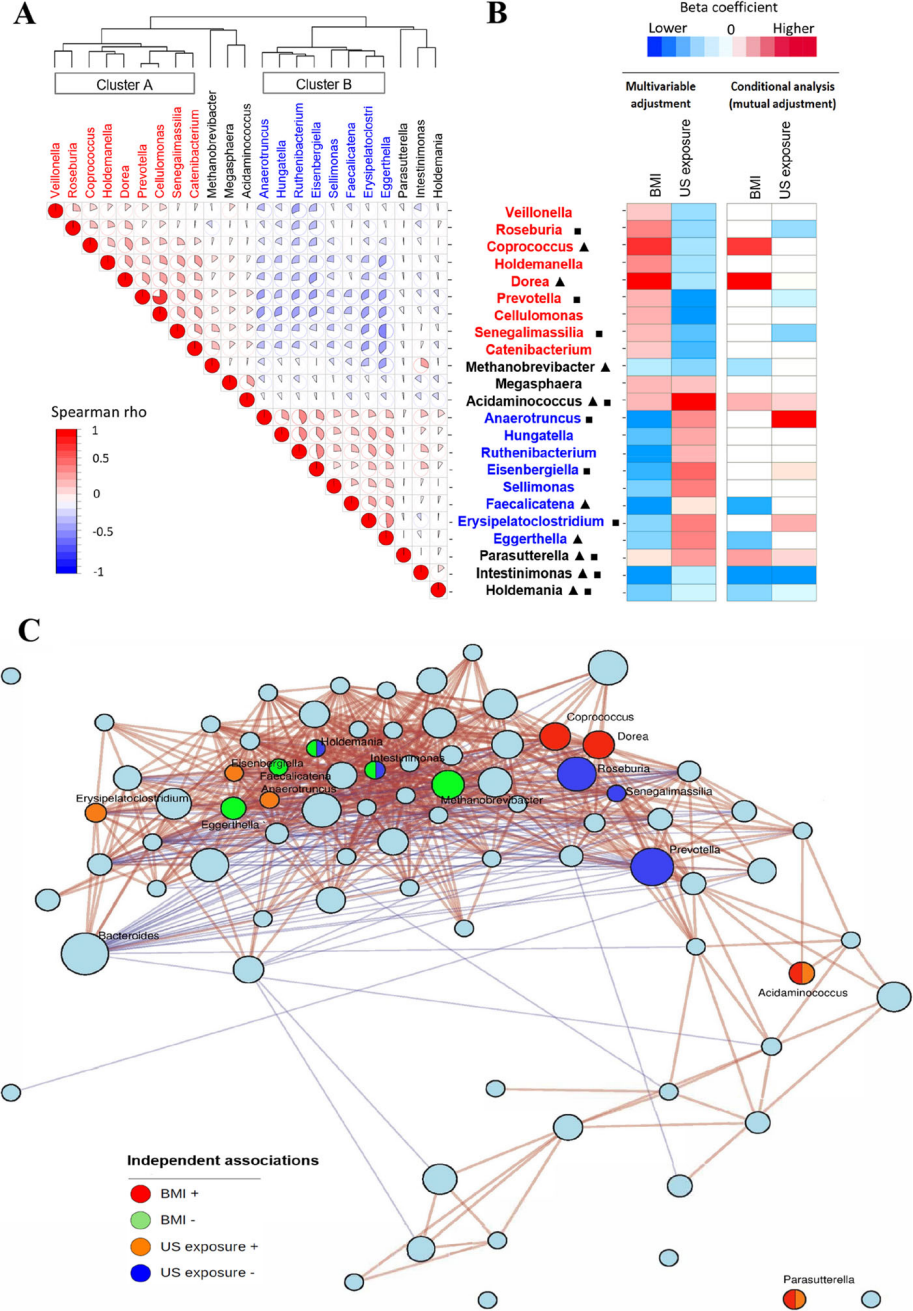
Relationships among the identified gut microbial genera associated with both obesity and US exposure. **A** Spearman correlation heatmap for the 23 identified bacterial genera associated with both obesity and US exposure. **B** Associations of 23 gut bacteria genera with obesity and US exposure. These associations were estimated in linear regression models after adjustment for demographic, socioeconomic, behavioral, and clinical factors (multivariable adjustment, FDR *P* < 0.05 level) and further adjustment for other bacterial genera (conditional analysis, mutual adjustment). Red/blue colors depict significant positive/inverse associations and the gradient colors reflect the ranks of effect sizes estimated in linear regression models, while white color indicates non-significant associations. Triangle and square indicate the microbial genera that were independently associated with obesity and US exposure after mutual adjustment, respectively. **C** Co-occurrence network representing the structure of microbial composition. The size of node reflects the cumulative sum scaling normalized abundance of each taxon. Red/green nodes indicate genera positively/inversely associated with host obesity after mutual adjustment (*P* < 0.05), and orange/ blue nodes indicate genera positively/inversely associated with US exposure after mutual adjustment (*P* < 0.05). Positive (red lines) and inverse (blue lines) correlations were obtained between predominant genera using Spearman rho based co-occurrence network analysis

We then examined correlations among these 23 genera associated with both obesity and US exposure. Given the compositional data, we applied multiple methods including Spearman, Pearson and SparCC to calculate correlation coefficients among bacteria genera [31], which showed generally consistent results (Additional file 1: Figure S5). As gut microbiome data tend to be zero-inflated, this may influence the correlation estimates. Thus, we performed sensitivity analyses after excluding samples with absent bacteria genera, and similar correlations among these genera were observed (Additional file 1: Figure S5). The 17 bacterial genera which showed paradoxical associations formed two clusters which were inversely correlated (Fig. 2A). Cluster A included correlated bacteria (Spearman *r* = 0.06–0.77) that were associated with higher BMI and less US exposure. Cluster B included correlated bacteria (Spearman *r* = 0.18–0.46) that were associated with lower BMI and greater US exposure.

Given the moderate correlations among many of these genera, we hypothesized that the observed paradoxical associations might be due to correlations/co-occurrence among these genera. To test this hypothesis, we performed conditional analyses including all genera associated with both BMI and US exposure in the same model, except for *Cellulomonas* which was excluded due to its high correlation with *Prevotella* (*r* = 0.77), to identify potentially independent bacterial taxa associated with obesity and/or US exposure. After controlling for other genera, nine genera remained significantly associated with obesity, and 10 genera remained significantly associated with US exposure (Fig. 2B, and Additional file 1: Table S11). No paradoxical associations remained after conditional analyses. Among six bacteria outside of clusters A and B, *Acidaminococcus* and *Parasutterella* were associated both with higher BMI and greater US exposure, while *Intestinimonas* and *Holdemania* were associated with lower BMI and less US exposure. Among nine members of Cluster A, *Coprococcus* and *Dorea* were associated with higher BMI only, and *Roseburia*, *Prevotella* and *Senegalimassilia* were associated with less US exposure only. Among eight members of Cluster B, *Faecalicatena* and *Eggerthella* were associated with lower BMI only, and *Anaerotruncus*, *Eisenbergiella*, and *Erysipilatoclostria* were associated with greater US exposure only. To minimize potential influences of over-adjustment, we conducted conditional analyses which only included four independent BMI-associated genera (*Coprococcus*, *Dorea*, *Faecalicatena*, and *Eggerthella*) in the models. The associations of other 13 genera in clusters A or B with BMI were not significant after adjustment for these four independent BMI-associated genera (Additional file 1: Table S11). Similarly, the associations of other 11 genera in cluster A or B with US exposure were not significant after adjustment for six independent US exposure-associated genera. In addition, we examined the associations of these genera with two other obesity measurements, body fat percentage, and waist circumference (Additional file 1: Figure S6), as well as severity of obesity (normal weight, 18.5 ≤ BMI < 25 kg/m^2^; overweight, 25 ≤ BMI < 30 kg/m^2^); obese I, 30 ≤ BMI < 35 kg/m^2^; obese II, 35 ≤ BMI < 40 kg/m^2^; and obese III, BMI ≥ 40 kg/m^2^). The results on the associations of these genera with body fat percentage, waist circumference, and severity of obesity in the conditional analyses were generally consistent with those on the associations between these genera and BMI (Additional file 1: Figure S6, and Additional file 1: Table S12).

In order to explore the relationship among microbial genera in a compositionally coherent manner, we also applied differential ranking analysis using songbird pipeline [32] in which the natural log-ratio of microbial genera data was calculated using *Clostridium* as the reference. We built a Spearman correlation heatmap using these log-ratio data (Additional file 1: Figure S6), and the correlations among microbial genera were generally consistent with those estimated using the CSS transformed data. For examples, we also observed significant positive correlations among *Coprococcus*, *Roseburia*, *Prevotella*, and *Dorea*, suggesting potential bacterial co-occurrence of these genera.

We also performed co-occurrence network analysis [33, 34] to visualize potential bacterial co-occurrence relationships among gut bacterial genera in our study samples. This analysis indicated potential bacterial co-occurrence relationships among these genera which showed associations with both obesity and US exposure (Fig. 2C), and their associations might be driven by some key genera associated with obesity or US exposure. For example, among Cluster-A bacterial genera which showed bacterial co-occurrence relationships, *Coprococcus* and *Dorea* were associated with higher BMI, while *Roseburia*, *Prevotella*, and *Senegalimassilia* were associated with less US exposure. Interestingly, either phylogenetically closely related bacterial genera, or those belonging to different phyla, could be clustered in the bacterial co-occurrence network. For example, *Roseburia, Coprococcus*, and *Dorea* are in the *Lachnospiraceae* family, while *Prevotella and Senegalimassilia* belong to the *Bacteroidetes* phylum and the *Actinobacteria* phylum, respectively (Fig. 1A). Taken together, these results suggested that the observed associations of individual gut microbial taxa with obesity and US exposure might be due to bacterial co-occurrence, and these paradoxical associations were resolved after taking bacterial co-occurrence into account.

### US exposure, dietary intake, and gut microbiome

**Fig. 3:**
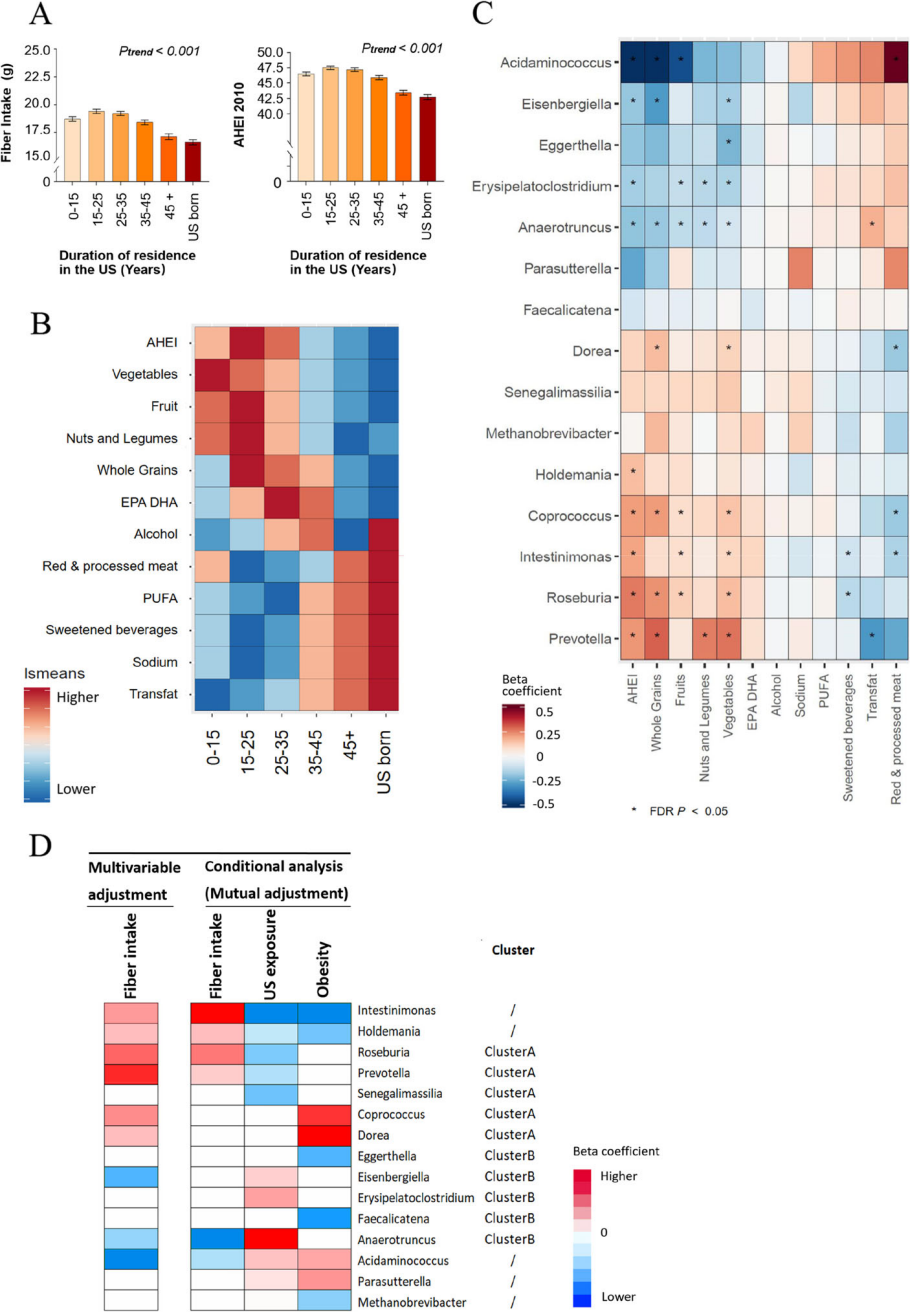
US exposure, dietary intake, and gut microbial genera. **A** Associations of US exposure with dietary fiber intake and AHEI-2010 score. Data are adjusted means (SEs) of dietary fiber intake and AHEI-2010 score across US exposure groups estimated in linear regression models after adjustment for demographic, socioeconomic, behavioral, and clinical factors. **B** Heatmap for overall dietary quality and individual dietary factor associated with US exposure. Gradient colors reflect the ranks of adjusted means of AHEI-2010 score and 11 food/nutrition component of AHEI-2010 across US exposure groups estimated in linear regression models after adjustment for demographic, socioeconomic, behavioral, and clinical factors. **C** Heatmap for overall dietary quality and individual dietary factor associated with gut microbial genera. This heatmap includes 15 microbial genera that were independently associated with obesity and/or US exposure. Data are effect sizes of dietary factors on gut microbial genera estimated in linear regression models after adjustment for demographic, socioeconomic, behavioral, and clinical factors. **D** Associations of gut microbiota with dietary fiber intake, US exposure, and host obesity. These associations were estimated after adjustment for demographic, socioeconomic, behavioral, and clinical factors (multivariable adjustment, FDR *P* < 0.05 level) and further adjustment for other bacterial genera (conditional analysis, mutual adjustment). Red/blue colors depict significant positive/inverse associations and the gradient colors reflect the ranks of effect sizes estimated in linear regression models, while white color indicates non-significant associations

To further clarify the relationships of individual gut microbial taxa with obesity and US immigration, we attempted to account for diet, a behavioral factor that tends to change after immigration and that can profoundly influence both body habitus and gut microbiome features [11]. Compared to US-born Hispanics/Latinos, Latin America-born individuals, especially those who had relative shorter duration of US residence, showed higher consumption of dietary fiber, and generally more favorable eating habits, as evidenced by higher levels of the 2010-AHEI as well as individual food/nutrient components (e.g., higher intakes of vegetables, fruits, nuts, and legumes, and lower intakes of red/processed meat, sugar-sweetened beverages, sodium, and trans-fat) (Fig. 3A, B).

We then related individual bacteria genera with host dietary intake and found that 30 out of 84 predominant bacteria genera were significantly associated with 2010 -AHEI as well as individual food/nutrient components, especially with healthy fiber-rich foods (e.g., whole grain, vegetables, fruits, nuts, and legumes) (Additional file 1: Figure S8). Consistent with the relationship between US exposure and host dietary intake, bacterial genera associated with less US exposure were associated with relatively healthier diet pattern and higher intakes of fiber-rich foods, while bacterial genera associated with greater US exposure were associated with relatively unhealthier diet pattern and lower intakes of fiber-rich foods, regardless of their associations with obesity (Fig. 3C). We then focused on the associations of these bacteria genera with dietary fiber intake and found some unexpected associations. Particularly, *Dorea* and *Coprococcus*, which were independently associated with obesity, were associated with higher fiber intake (Fig. 3D). After including all 15 bacteria genera in a conditional analysis model (mutual adjustment) on fiber intake, no paradoxical associations remained. Six bacteria genera were independently associated with dietary fiber intake, and directions of these associations were in line with those of their associations with US exposure and/or obesity (Fig. 3D).

### US exposure, fiber intake, and gut microbiome functional components

Lastly, we examined microbial function contents to better understand the interrelationship among US immigration, dietary intake, gut microbiome, and obesity. The gut microbiome functional profiles were obtained using SHOGUN [35] and annotated by the Kyoto Encyclopedia of Genes and Genomes (KEGG) database Release 94.0 [36], and a total of 1952 known enzymes with specific Enzyme Commission numbers (EC number) were identified and included in the analyses. We first examined associations between US exposure and KO groups for 1952 known enzymes using weighted linear regression models and identified 260 KO groups associated with US exposure (all FDR *P* < 0.1). We then performed an enrichment test for these KO groups at EC level II enzyme category and found an enrichment of enzymes belonging to the glycosylases associated with US exposure (Additional file 1: Table S13).

**Fig. 4:**
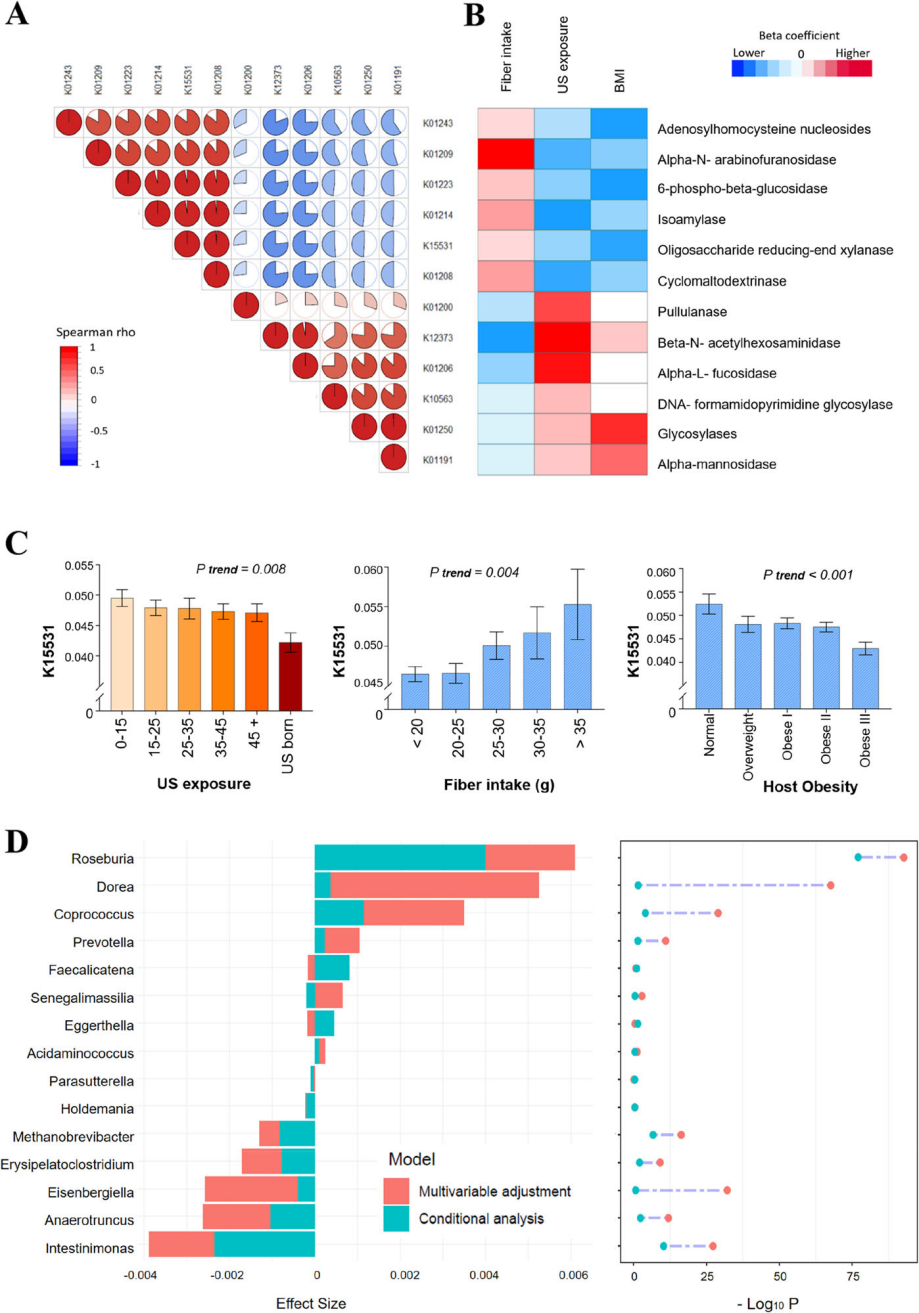
Gut microbial functional components associated with both US exposure and dietary fiber intake, and their associations with obesity and gut bacterial genera. **A** Correlation heatmap for 12 microbial functional enzymes associated with both US exposure and dietary fiber. **B** Association of 12 microbial functional enzymes with US exposure, dietary fiber intake and BMI. These associations were estimated in linear regression models after adjustment for demographic, socioeconomic, behavioral, and clinical factors. Red/blue colors depict significant positive/inverse associations (FDR *P* < 0.05) and the gradient colors reflect the ranks of effect sizes estimated in linear regression models, while white color indicates non-significant associations. **C** Levels of gut microbial enzyme K15531 xylanase according to US exposure, dietary fiber intake, and host obesity. Data are adjusted mean (SE) of enzyme K15531 xylanase (centered log-ratio transformed) across US exposure groups, levels of dietary fiber intake, and host obesity status estimated in linear regression models after adjustment for demographic, socioeconomic, behavioral, and clinical factors. **D** Associations of 15 gut bacterial genera with gut microbial enzyme K15531 xylanase. Data are effect sizes and *P* values estimated in linear regression models after adjustment for demographic, socioeconomic, behavioral, and clinical factors (multivariable adjustment) and further adjustment for other bacterial genera (conditional analysis, mutual adjustment)

We thus focused on the glycosylases and found 12 KO groups associated with US exposure (all FDR *P* < 0.1, Fig. 4A; and Additional file 1: Table S14). These KO groups formed two clusters and KO groups within each cluster were highly correlated with each other (Fig. 4A). The first cluster which was associated with less US exposure included KO groups encoding enzymes related to degradation of dietary fibers. For example, oligosaccharide reducing-end xylanase (K15531; EC3.2.1.156) was known as a high molecular mass xylanases which can degrade xylan, a type of dietary fiber found in plant cell walls [37, 38]. Consistently, these KO groups were associated with higher fiber intake. The second cluster which was associated with greater US exposure included KO groups encoding enzymes related to starch degradation (e.g., Pullulanase, K01200, EC3.2.1.41) or carbohydrate metabolism associated with obesity and insulin resistance (e.g., alpha- mannosidase, K01191, EC3.2.1.24) [39]. These results suggest that, compared to those with a shorter duration of US residence, US-born Hispanics/Latinos and those with a longer duration of US residence may have gut microbiota with decreased capacity for fiber degradation and increased capacity for starch degradation and simple carbohydrate utilization, potentially related to decreased consumption of fiber-rich foods and increased consumption of refined grains and sugary foods. We then related these KO groups with BMI and found expected associations showing that all KO groups associated with less US exposure and higher fiber intake were associated with lower BMI, and some KO groups associated with greater exposure and lower fiber intake were associated with higher BMI (Fig. 4B). For example, levels of oligosaccharide reducing-end xylanase (K15531) deceased with greater US exposure, increased with higher fiber intake, and deceased with higher BMI/severity of obesity (Fig. 4C). The inverse association between oligosaccharide reducing-end xylanase and US exposure might be mainly driven by relatively lower levels of oligosaccharide reducing-end xylanase in US-born Hispanics/Latinos compared to immigrant Hispanics/Latinos, as we only found a trend for the inverse association between oligosaccharide reducing-end xylanase and years of US residence among immigrant Hispanics/Latinos (*P* = 0.10).

We then explored potential contributions of these 15 bacterial genera that were independently associated with US exposure, fiber intake, and/or obesity to these KO groups. As these KO groups were highly correlated with each other, we focused on xylanase (K15531) as an example, given its well-known biological function in fiber degradation. While we found expected positive associations between bacterial genera (e.g., *Roseburia and Prevotella* which were associated with higher fiber intake) and xylanase, *Dorea* and *Coprococcus* which were positively associated with obesity were also positively associated with xylanase (Fig. 4D). We performed conditional analysis by including all 15 genera in the same regression model on xylanase. This greatly attenuated or abolished most of the associations of these genera, especially *Dorea* and *Coprococcus*, with xylanase, while the association between *Roseburia* and xylanase was only slightly attenuated and remained the strongest (Fig. 4D). These results suggest that *Roseburia* might be a major bacteria contributing to xylanase, and the observed positive associations of *Dorea* and *Coprococcus* with xylanase might be due to their correlations with *Roseburia*. Our genomic analyses provided further evidence supporting the presence of xylanase gene on representative *Roseburia* genomes but not on representative *Dorea* or *Coprococcus* genomes (Additional file 1: Table S15). In support of our results, *Roseburia* species from the human gut have been reported to produce xylanases and display high xylanolytic activity [38].

## Discussion

Taken together, these data from a large population-based cohort study of Hispanics/Latinos living in the US demonstrated that US immigration was associated with alterations in gut microbiome diversity, individual taxa, and functional profiles, which is consistent with previous findings observed in immigrants from Thailand to the USA [10]. Further extending prior work, this study linked these US immigration-associated gut microbiome features with obesity, a major health burden among US Hispanics/Latinos and other US immigrants from lower-to-middle income countries [12]. In particular, we focused on individual gut microbial taxa associated with both US immigration and obesity. We identified several gut bacterial genera associated with US immigration, host dietary intake, and obesity in expected directions, suggesting a potential involvement of these bacteria linking US immigration with obesity, though potential mechanisms underlying these associations remain unknown. For example, *Acidaminococcus* which was associated with obesity in the current study and other populations [40, 41] was found to be more abundant in US-born Hispanics/Latinos and those with a longer duration of US residence, compared to those with a shorter duration of US residence, potentially related to increased consumption of animal-based protein-rich foods (e.g., red/processed meat intake; Fig. 3B), since *Acidaminococcus* is known to use amino acids as energy source [42]. However, it remains unknown how increased gut *Acidaminococcus* may influence host obesity.

Another major contribution of this study is to demonstrate that considering potential microbial co-occurrence may help clarify the paradoxical associations of individual gut microbial taxa with US immigration and obesity. Our current analysis suggested that the observed associations of some gut microbioal taxa with US immigration or obesity might be due to their co-occurrence with other taxa. For example, we found that *Roseburia* along with *Prevotella* were the most common genera associated with US immigration in this population, and their paradoxical associations with obesity might be due to their correlations with *Dorea* and other key obesity-associated taxa. Unlike *Prevotella*, the influence of immigration on *Roseburia* was not reported before, although *Roseburia* is known to degrade and use xylans and other dietary fibers [38, 43, 44] and the potential beneficial role of *Roseburia* in health has also been suggested [43, 45]. Our data indicate that *Roseburia*, like *Prevotella*, might be another gut microbiome signature of non-industrialized populations and might decrease during westernization. On the other hand, the paradoxical association between *Dorea*, an obesity-associated bacterial genus in ours and other studies [46, 47], and less US exposure might be related to *Roseburia* and other key taxa associated with US immigration. Although it is unknown how *Dorea* may influence host obesity, our data suggested that this might not be related to US immigration.

It has been suggested that high strain-level diversity of *Prevotella* might explain the inconsistent associations of *Prevotella* with human health and disease across populations [22, 23]. However, there are scant data on *Prevotella* strains in relation to human disease traits given the difficulties of strain-level analysis in large human population-based studies. A recent study reported that *Prevotella copri*, the most abundant species of *Prevotella* in the human gut, may encompass four distinct clades, but there was little evidence suggesting these four clades to be differentially associated with obesity, diabetes, or other human diseases [21]. Our study provides another potential explanation. The inconsistent associations between *Prevotella* and human diseases (e.g., obesity) might be due to variation in correlations between *Prevotella* and some true disease-associated taxa across populations. For example, the observed association between *Prevotella* and obesity in this US Hispanic/Latino population might be due to the correlation between *Prevotella* and *Dorea*, an obesity-associated bacterial genus. However, more studies are needed to clarify whether the inconsistent associations between *Prevotella* and human diseases are due to strain-level diversity of *Prevotella*, correlations between *Prevotella* and disease-associated bacteria, or both.

The observed bacterial co-occurrence relationships might reflect some positive bacterial interactions that generate increased benefits for the group, such as mutualism (e.g., antibiotic resistance conferral), commensalism (e.g., cross-feeding on compounds produced by other members), and synergism (e.g., syntrophic cooperation) [9]. Our results are also supported by the previously reported co-occurrence relationship between *Coprococcus* and *Roseburia*, both in the *Lachnospiraceae* family [34, 48]. These data suggest that the phylogenetically closely related genera could also have co-occurrence relationship rather than always compete against each other due to their similar habitat preference [34]. However, due to the poor mechanistic understanding of the human gut microbial community, the ecological links among gut bacteria should be interpreted with caution. Although the underlying mechanisms for the bacterial co-occurrence network need to be elucidated, our findings may have important implications in the future studies investigating associations of gut microbiome with human health and disease. We have shown that the associations of individual taxa with environmental factors (e.g., US exposure) and host phenotypes (e.g., obesity) could be confounded by other taxa due to potential bacterial co-occurrence, and thus, conditional analysis using mutual adjustment model is needed to examine the independent associations of individual taxa with environmental factors and host phenotypes. Furthermore, microbial co-occurrence network analysis [28] might be a useful approach to examine the bacterial co-occurrence relationship and its potential influences on the associations between gut microbiome and human health and disease.

This study has several limitations. Data on diet were assessed prior to the gut microbiome assessment, although we obtained dietary information using rigorous methods based on two 24-h dietary recalls and a food propensity questionnaire designed to capture habitual long-term diet [49] and detected strong associations between host dietary intake and gut microbiome features. The potential urban-rural differences could not be evaluated in our study due to the lack of detailed information on place of origin. Due to limitations of shallow shotgun metagenomics, we were unable to examine strain-level data and determine individual taxa contributing to gut microbiome functional components using SHOGUN pipeline [35]. Given the observational nature of this study, causal inference could not be established without further evidence.

## Conclusions

In conclusion, this study in US Hispanics/Latinos found that US immigration was associated with reduced gut microbiome diversity, reduced gut microbiome functions of fiber degradation, and alterations in individual gut microbial taxa (e.g., increased *Acidaminococcus*, *decreased Roseburia*, and *Prevotella*), potentially related to westernized diet during acculturation process. The US immigration-related alterations in gut microbiome features were also associated with obesity, suggesting a potential role of gut microbiome in the development of obesity among US immigrants from Latin America. Of note, a majority of gut bacterial genera showed paradoxical associations with US immigration, host dietary intake, and obesity, which could be resolved into directionally consistent associations after accounting for potential microbial co-occurrence. Thus, potential microbial co-occurrence may be an important factor to consider in future studies relating individual gut microbial taxa to environmental factors and host health and disease.

## Methods

### Study population

HCHS/SOL is a prospective, population-based cohort study of 16,415 Hispanic/Latino adults (aged 18–74 years at the time of recruitment during 2008–2011) who were selected using a two-stage probability sampling design from randomly sampled census block areas within four US communities (Chicago, IL; Miami, FL; Bronx, NY; San Diego, CA) [11, 29, 30]. A comprehensive battery of interviews and a clinical assessment with blood draw were conducted at in-person clinic visits. Information on demographics, behaviors, health status, family and medical histories, and medication use was collected using structured questionnaires, and blood pressure and anthropometric traits were measured. Blood biomarkers including blood lipids and glycemic traits were measured by standard methods [50]. The HCHS/SOL Gut Origins of Latino Diabetes (GOLD) ancillary study was conducted to examine the role of gut microbiome in the development of multiple health outcomes, enrolling 3035 participants from the HCHS/SOL approximately concurrent with the second in-person visit period from 2014 to 2017 [11]. This study included participants of diverse Hispanic/Latino backgrounds, including Dominican, Cuban, Puerto Rican, Mexican, Central American, and South American. The study was approved by the institutional review boards of corresponding site institutions. Written informed consent was obtained from all participants.

### Fecal specimen collection and Shotgun metagenomics sequencing

Enrolled GOLD participants were provided with a stool collection kit. For each participant, a single fecal specimen was self-collected using a disposable paper inverted hat (Protocult collection device, ABC Medical Enterprises, Inc., Rochester, MN). Two fecal samples were self-collected by each participant, with one sample collected by a Whatman FTA card (GE Healthcare, Chicago, IL) and another one placed in a tube of RNA*later* (Invitrogen, Carlsbad, CA). Detailed procedures have been described elsewhere [11]. In addition, use of antibiotics or probiotic supplements within the prior 6 months and stool characteristics were ascertained by self-administered questionnaire at the time of stool sample collection.

Metagenomics Sequencing was performed on DNA extracted from fecal samples collected by FTA card using a novel shallow-coverage method of shotgun sequencing-based Illumina NovaSeq platforms [51]. The adapters and barcode indices are processed following the iTru adapter protocol [52]. De-multiplexing was applied to generate Shallow shotgun per-sample FASTQ data and the adapter sequences were trimmed. The human-filtered FASTQ reads were further trimmed to remove low-quality bases that had a PHRED quality score of 25 or less using prinseq-lite 0.20.4 (https://edwards.sdsu.edu/cgi-bin/prinseq/prinseq.cgi) [53]. For the final analytical set, two Illumina sequencing runs were pooled. Samples with a coverage depth less than 500,000 reads per sample were excluded. Of 3035 samples, 2640 samples passed all QC metrics and were used in the current analysis. The coverage depth ranged from 500 to 8945 k reads per sample and average depth was 955 k reads per sample. The quality-controlled paired end data was then concatenated and aligned against the NCBI RefSeq representative prokaryotic genome collection (release 82) [54] using default SHOGUN [35] settings. Bowtie2 [55] was selected as the alignment tools in SHOGUN pipeline (https://github.com/knights-lab/SHOGUN). The reads that mapped to a single reference genome is labeled with the NCBI taxonomic annotation at the species level. Those reads that mapped to multiple reference genomes are labeled as the last common ancestor (LCA) of each label according to the NCBI taxonomy [35]. The α-diversity indices (Faith’s phylogenetic distance), and β-diversity weighted UniFrac distances were calculated using Qiita (https://qiita.ucsd.edu/) [56], Metaphlan3(https://huttenhower.sph.harvard.edu/metaphlan/) [57], and R phyloseq / vegan packages (https://github.com/joey711/phyloseq/https://github.com/vegandevs/vegan) [58, 59]. Functional profiles were obtained using SHOGUN and the Kyoto Encyclopedia of Genes and Genomes (KEGG) database release 94.0 [36].

### Assessment of US exposure, obesity, and dietary intake

Information on Hispanic/Latino background, place of birth, and years living in the mainland USA (with the US territory of Puerto Rico considered to be part of Latin America) was collected using structured questionnaires [11, 29, 30]. US exposure was defined based on place of birth and duration of US residence: mainland US born, US residence of 0–< 15 years, US residence of 15–< 25 years, US residence of 25–< 35 years, US residence of 35–< 45 years, and US residence of ≥ 45 years. Body mass index (BMI) was calculated as measured weight (kg) divided by measured height squared (m^2^). Obesity status was defined by BMI: normal weight (18.5 ≤ BMI < 25 kg/m^2^), overweight (25 ≤ BMI < 30 kg/m^2^), obese I (30 ≤ BMI < 35 kg/m^2^), obese II (35 ≤ BMI < 40 kg/m^2^), and obese III (BMI ≥ 40 kg/m^2^). Other obesity measures included waist circumference, and body fat percentage obtained from the Tanita body composition analyzer (model TBF-300A; Tanita Corporation, Arlington Heights, IL).

Usual dietary intake was estimated using the National Cancer Institute methodology based on dietary data collected from two 24-h dietary recalls and a food propensity questionnaire (FPQ), as described previously [60]. In brief, the first dietary recall was administered through in-person interviews conducted at the time of the baseline visit, whereas the second was performed primarily via telephone approximately 30 days after the first interview. The FPQ, which was administered at the 1-year follow-up call, asked participants to report frequencies of foods eaten in the previous year. Foods and nutrients were analyzed using the multiple-pass methods of the Nutrition Data System for Research software (version 11) from the Nutrition Coordinating Center at University of Minnesota. Overall dietary quality for each participant was estimated by the 2010 Alternative Healthy Eating Index (AHEI-2010) which was established based on extensive epidemiologic findings linking foods and nutrients to chronic disease outcomes [61]. The AHEI-2010 consists of 10 food/nutrient components in addition to alcohol consumption, with each of the 11 components being given 0–10 points according to predetermined criteria.

### Statistical analysis

Clinical and demographic characteristics of the study population were described by reporting means with standard deviations (SDs) or medians with interquartile ranges for continuous variables and absolute frequencies with percentages for categorical variables.

Weighted linear regression models were applied to examine associations of microbial α-diversity indices (Faith’s phylogenetic distance) with BMI and US exposure, adjusting for age, sex, study center, education level, income, antibiotic use in the last 6 months, moderate-to-vigorous physical activity, total energy intake, diabetes, and metformin use. Permutational multivariate analysis of variance (PERMANOVA) and principal-coordinate analysis (PCoA) were carried out with weighted UniFrac distances for the microbial β-diversity analyses.

For metagenomics taxonomic analyses, a total of 84 predominant bacterial genera with average relative abundance ≥0.01% were included (a total of 596 species belonged to these predominant genera). Cumulative sum scaling (CSS) normalization was conducted [62] for each of taxonomic variables before analyses. Associations of bacterial taxonomic features with BMI and US exposure were examined using weighted multivariable linear regression, with adjustment for the aforementioned covariates. Furthermore, we further adjusted for Hispanic background in additional multivariable regression models. All the models incorporated the sampling weights from the complex survey design of the HCHS/SOL [29, 30]. We constructed integrated hierarchical tree using iTol (https://itol.embl.de/) [63].

In order to explore the relationship among microbial genera in a compositionally coherent manner, we applied differential ranking (DR) analysis using songbird pipeline (https://github.com/biocore/songbird ) [32]. Read counts were summarized at the genus level and inputted into Songbird [32] for multinomial regression. To ensure proper model fit while guarding against potential model overfitting, a null model was generated using similar parameters. Comparing this null model to the fitted model demonstrated better fit for the latter (pseudo-Q2 = 0.022301), enabling further utilization of the differentials [32]. These differentials were then inputted into Qurro for visualization [64]. Then we selected the genus *Clostridium* as the reference, since it was stable (with minimum absolute ranks) across experimental conditions (BMI) and present across most samples. The natural log-ratio of microbial genera data was calculated using *Clostridium* as the reference and we also built Spearman correlation heatmap using these log-ratio data.

We examined associations of bacterial genera with BMI and US exposure stratified by sex, age group dichotomized at the median age of study participants (age < 55 or ≥ 55 years), study center (Chicago, Miami, Bronx, or San Diego), Hispanic/Latino background (6 strata: Dominican, Cuban, Puerto Rican, Mexican, Central American, and South American; 2 strata: Mainland or Caribbean), place of birth (US born or non-US born; BMI analysis only), and obesity status (obese or non-obese; US exposure analysis only). We also examined the associations of bacterial genera with obesity and US exposure among the first-generation Hispanic/Latino immigrants according to the place of birth (i.e., Dominica, Cuba, Puerto Rica, Mexico, Central America, and South America). In addition, we performed sensitivity analyses by excluding individuals with antibiotic use during the past 6 months.

To identify potential independent bacterial genera associated with BMI or US exposure, we included all 23 genera which were associated with both BMI and US exposure in the same regression model on BMI or US exposure (mutual adjustment, conditional analysis). We applied multiple methods including Spearman, Pearson, and SparCC to calculate correlation coefficients among bacteria genera [31]. In addition, we performed hierarchical clustering approach to identify the clusters. Microbial co-occurrence network, which infer ecological associations based on taxonomic composition data obtained from high-throughput sequencing techniques [28], are widely used to visualize the statistically significant associations between microbes in microbial communities and predict the potential microbe interactions [9]. In this study, we performed Spearman rho based co-occurrence network analysis with Cytoscape (https://cytoscape.org/) [33], to explore the relationships among the identified bacterial taxa and to better describe the overall structure of microbial components. Nodes and edges represent bacterial genera and statistically significant correlations between these genera, respectively.

Associations of dietary fiber intake, AHEI-2010 score, and 11 food and nutrient components of AHEI-2010 with US exposure and 84 predominant gut bacterial genera were examined using weighted multivariable linear regressions, with adjustment for the aforementioned covariates. The conditional analysis with mutual adjustment further included 15 identified genera which were independently associated with BMI and/or US exposure, to further examine independent associations between bacterial taxa and dietary fiber intake.

For metagenomics functional analyses, centered log-ratio transformation was applied to the KEGG ortholog group abundances. Weighted linear regression models were applied to examine associations of KEGG ortholog groups with US exposure, dietary fiber intake, and BMI, after controlling for the aforementioned covariates, and incorporating the sampling weights from the complex survey design. An enrichment analysis using Fisher’s exact test was performed for the 1952 annotated enzymes at EC level II enzyme category. Partial spearman correlation analysis was use to estimate correlation coefficients between bacterial genera (CSS normalized) and KO groups (centered log-ratio transformed). Associations of 15 genera which were independently associated with obesity and/or US exposure with xylanase (K15531) were examined using linear regression models after multivariable adjustment. We further included all 15 genera in the same model (mutual adjustment, conditional analysis) to examine potential independent associations of these genera with xylanase (K15531).

The Benjamini-Hochberg false discovery rate (FDR) method was used for multiple testing correction. Statistical analyses were performed using R 3.6.1. unless otherwise stated.

## Supplementary Information

**Additional file 1: Figure S1.** Microbial community diversity associated with obesity and US exposure. **Figure S2.** Beta diversity PERMANOVA analysis for obesity, US exposure, and other co-variates, ranked by R^2^ **Figure S3.** Associations of gut microbiota and host obesity (BMI), stratified by: A. Sex; B. Age group (< 55 and > = 55 years); C. Place of birth (US born and non-US born); D. Hispanic origin (Caribbean [Dominican, Cuban, Puerto Rican] and Mainland [Central American, Mexican, South American]) ; E to J. Field Center (Bronx, Chicago, Miami, San Diego). **Figure S4.** Associations of gut microbiota and US exposure, further stratified by : A. Sex; B. Hispanic origin (Caribbean [Dominican, Cuban, Puerto Rican] and Mainland [Central American, Mexican, South American]); C. Obesity; D. Age group (< 55 and > = 55 years); E to J. Field Center (Bronx, Chicago, Miami, San Diego). **Figure S5.** Correlation heatmap for the 23 identified bacterial genera associated with both obesity and US exposure. **Figure S6.** Associations of 23 gut microbial genera with BMI, Body fat percentage and waist circumference. **Figure S7.** Correlation heatmap for all bacterial genera, using log ratios data and *Clostridium* as reference. **Figure S8.** Heatmap for overall dietary quality and individual dietary factor associated with gut microbial genera. **Table S1.** Characteristics of the study population by US exposure **Table S3.** Multivariable regression analyses: 23 genera associated with both obesity and US exposure. **Table S10.** Association of host obesity and US exposure with gut microbiota: species level analyses. **Table S11.** Conditional analysis for genera associated with both obesity and US exposure **Table S12.** Levels of gut microbial genera according to host obesity. **Table S13.** Target US exposure associated enzyme selection: Enrichment test for the annotated enzymes **Table S14.** Regression analyses: Enrichment test selected key enzymes associated with both US exposure and fiber intake. **Table S15.** The presence of key enzymes in specific bacteria: Alignment analysis.

**Additional file 2: Table S2.** Associations of 84 gut bacterial genera with obesity and US exposure.

**Additional file 3: Table S4.** Associations of 84 gut bacterial genera with obesity and US exposure after further adjusted for Hispanic background.

**Additional file 4: Table S5.** Associations of 84 gut bacterial genera with US exposure and all covariates in the multivariable regression model.

**Additional file 5: Table S6.** Associations of gut bacterial genera with obesity (BMI), stratified by Hispanic background groups.

**Additional file 6: Table S7.** Associations of gut bacterial genera with US exposure, stratified by Hispanic background groups.

**Additional file 7: Table S8.** Associations of gut bacterial genera with obesity (BMI), stratified by birth place, among the first generation immigrants.

**Additional file 8: Table S9.** Associations of gut bacterial genera with US exposure, stratified by birth place, among the first generation immigrants.

**Additional file 9.** Review history.

## Data Availability

HCHS/SOL data are archived at the National Institutes of Health repositories dbGap (accession number phs000810.v1.p1) and BIOLINCC (accession number HLB01141418a), and gut microbiome sequence data in this study are deposited in QIITA, ID 11666, and EMBL-EBI ENA, ERP117287 [65]. HCHS/SOL has established a process for the scientific community to apply for access to participant data and materials, with such requests reviewed by the project’s Steering Committee. These policies are described at https://sites.cscc.unc.edu/hchs/ (accessioned November 19, 2021). The corresponding author will accept reasonable requests for data and specimen access, which will be referred to the Steering Committee of the HCHS/SOL project.
