# Supplementary material for: Microbial co-occurrence complicates associations of gut microbiome with US immigration, dietary intake and obesity
Source: Genome Biol. 2021 Dec 10;22:336. doi: 10.1186/s13059-021-02559-w (PMC8665519; doi:10.1186/s13059-021-02559-w)
Supplement: Supplementary file 1 — Additional file 1: Figure S1. Microbial community diversity associated with obesity and US exposure. Figure S2. Beta diversity PERMANOVA analysis for obesity, US exposure, and other co-variates, ranked by R2 Figure S3. Associations of gut microbiota and host obesity (BMI), stratified by: A. Sex; B. Age group (< 55 and > = 55 years); C. Place of birth (US born and non-US born); D. Hispanic origin (Caribbean [Dominican, Cuban, Puerto Rican] and Mainland [Central American, Mexican, South American]) ; E to J. Field Center (Bronx, Chicago, Miami, San Diego). Figure S4. Associations of gut microbiota and US exposure, further stratified by : A. Sex; B. Hispanic origin (Caribbean [Dominican, Cuban, Puerto Rican] and Mainland [Central American, Mexican, South American]); C. Obesity; D. Age group (< 55 and > = 55 years); E to J. Field Center (Bronx, Chicago, Miami, San Diego). Figure S5. Correlation heatmap for the 23 identified bacterial genera associated with both obesity and US exposure. Figure S6. Associations of 23 gut microbial genera with BMI, Body fat percentage and waist circumference. Figure S7. Correlation heatmap for all bacterial genera, using log ratios data and Clostridium as reference. Figure S8. Heatmap for overall dietary quality and individual dietary factor associated with gut microbial genera. Table S1. Characteristics of the study population by US exposure Table S3. Multivariable regression analyses: 23 genera associated with both obesity and US exposure. Table S10. Association of host obesity and US exposure with gut microbiota: species level analyses. Table S11. Conditional analysis for genera associated with both obesity and US exposure Table S12. Levels of gut microbial genera according to host obesity. Table S13. Target US exposure associated enzyme selection: Enrichment test for the annotated enzymes Table S14. Regression analyses: Enrichment test selected key enzymes associated with both US exposure and fiber intake. Table S15. The presence of key [file 13059_2021_2559_MOESM1_ESM.pdf]

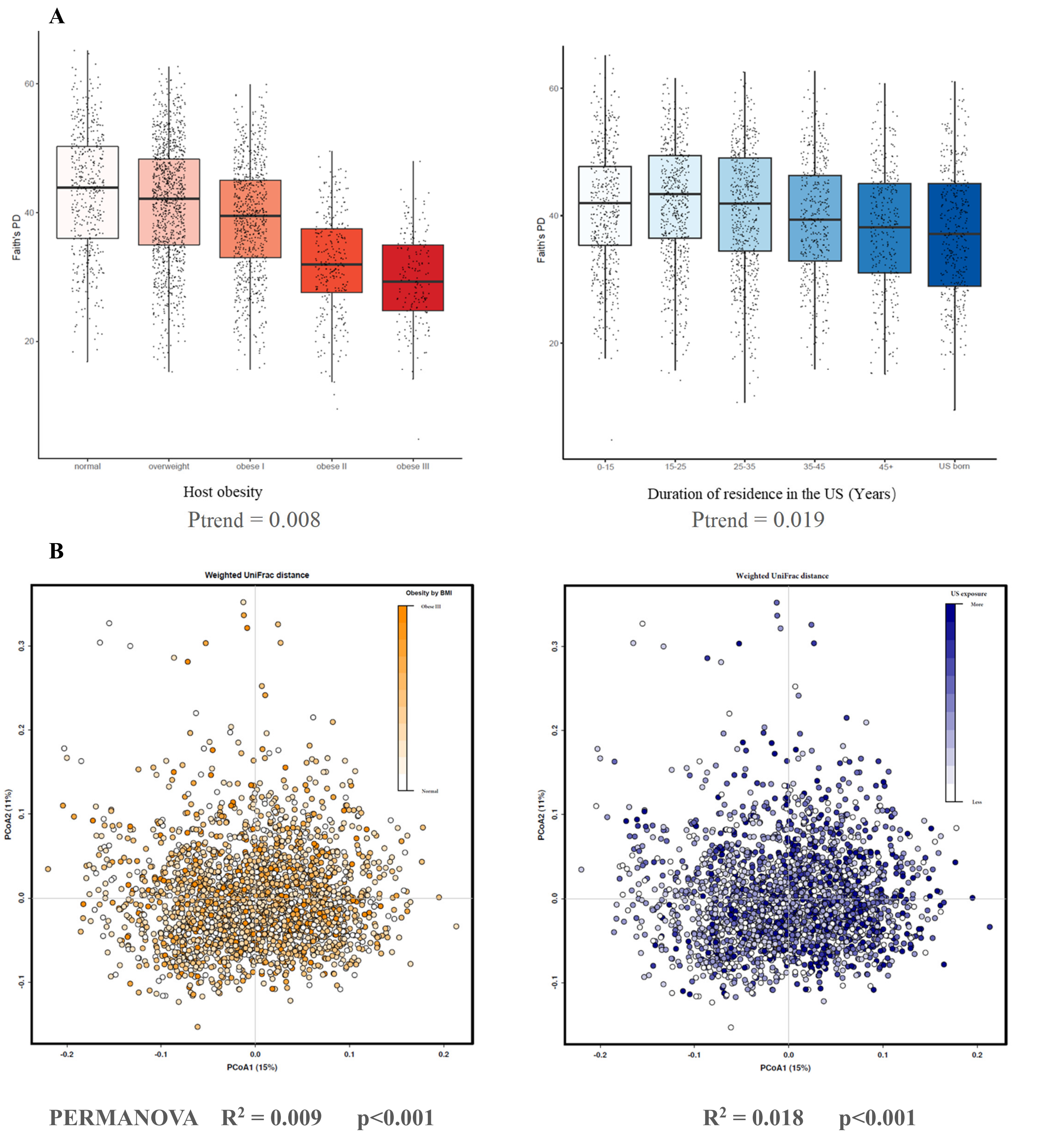

Figure S1. Microbial community diversity associated with obesity and US exposure. A. Associations of shotgun metagenomics alpha diversity Faith's phylogenetic distance (PD) with obesity and US exposure. These associations were estimated after adjustment for demographic, socioeconomic, behavioral and clinical factors. **B. Beta diversity Principal coordinate analysis based on weighted UniFrac distances.** Both of the host obesity and US exposure significantly co-varied with the diversity of the gut microbiome (P <0.001) and explained moderate proportion of variance. The adonis function from the vegan package was used to assess statistical significance for PERMANOVA.

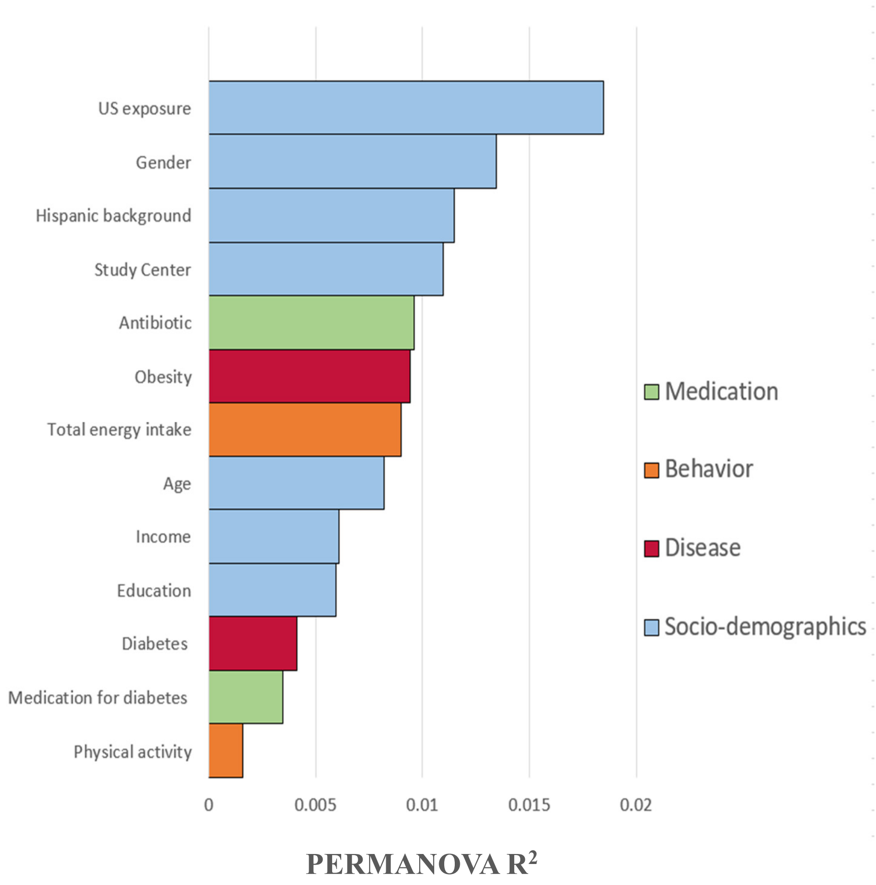

Figure S2. Beta diversity PERMANOVA analysis for obesity, US exposure, and other co-variates, ranked by R^2 . Calculated using the adonis function of the vegan package. All $P < 0.05$ except Physical activity.

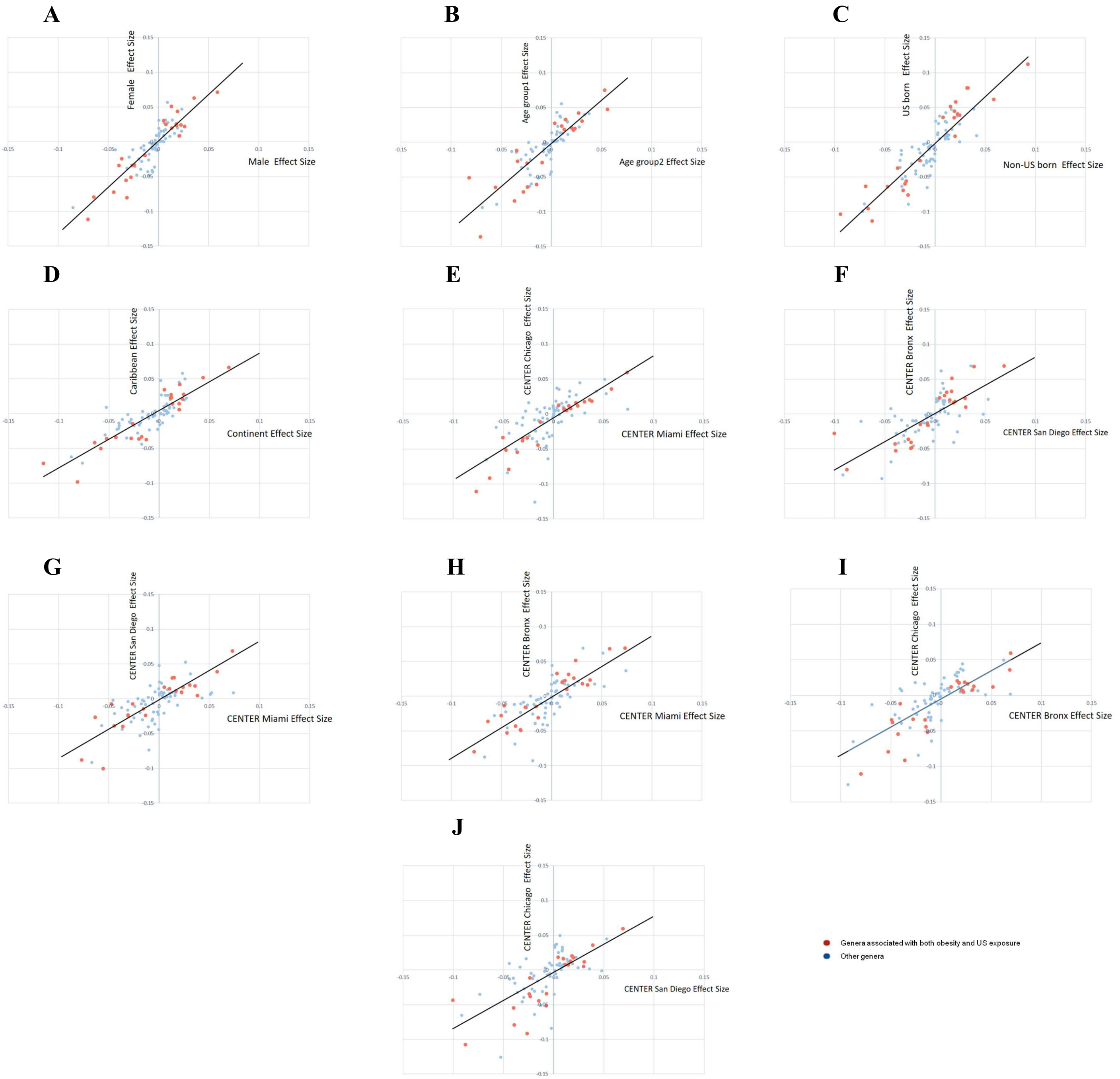

Figure S3. Associations of gut microbiota and host obesity (BMI), stratified by: A. Sex; B. Age group (< 55 and >= 55 years); C. Place of birth (US born and non-US born); D. Hispanic origin (Caribbean [Dominican, Cuban, Puerto Rican] and Mainland [Central American, Mexican, South American]) ; E to J. Field Center (Bronx, Chicago, Miami, San Diego). Each node represents one genus. All the predominant genera with average relative abundance >0.01% were included in this analysis. The 23 bacterial genera associated with both obesity and US exposure were highlighted by red, and no significant differences in associations between these 23 bacterial genera with BMI across strata were observed (all P for interaction >0/05). Models were adjusted for demographic, socioeconomic, behavioral and clinical factors.

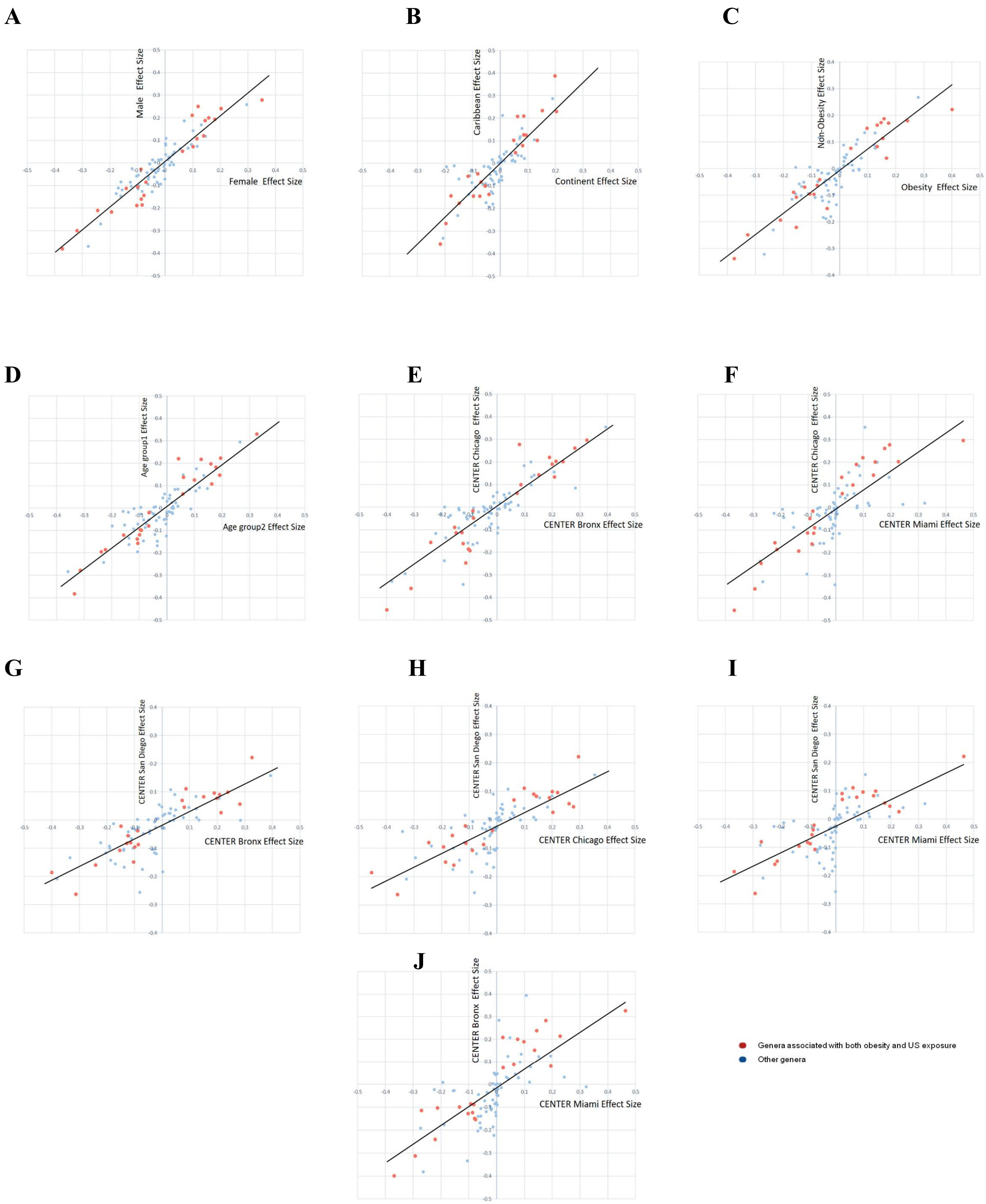

Figure S4. Associations of gut microbiota and US exposure, further stratified by : A. Sex; B. Hispanic origin (Caribbean [Dominican, Cuban, Puerto Rican] and Mainland [Central American, Mexican, South American]); C. Obesity; D. Age group (< 55 and >= 55 years); E to J. Field Center (Bronx, Chicago, Miami, San Diego). Each node represents one genus. All the predominant genera with average relative abundance >0.01% were included in this analysis. The 23 bacterial genera associated with both obesity and US exposure were highlighted by red. Models were adjusted for demographic and behavioral covariates.

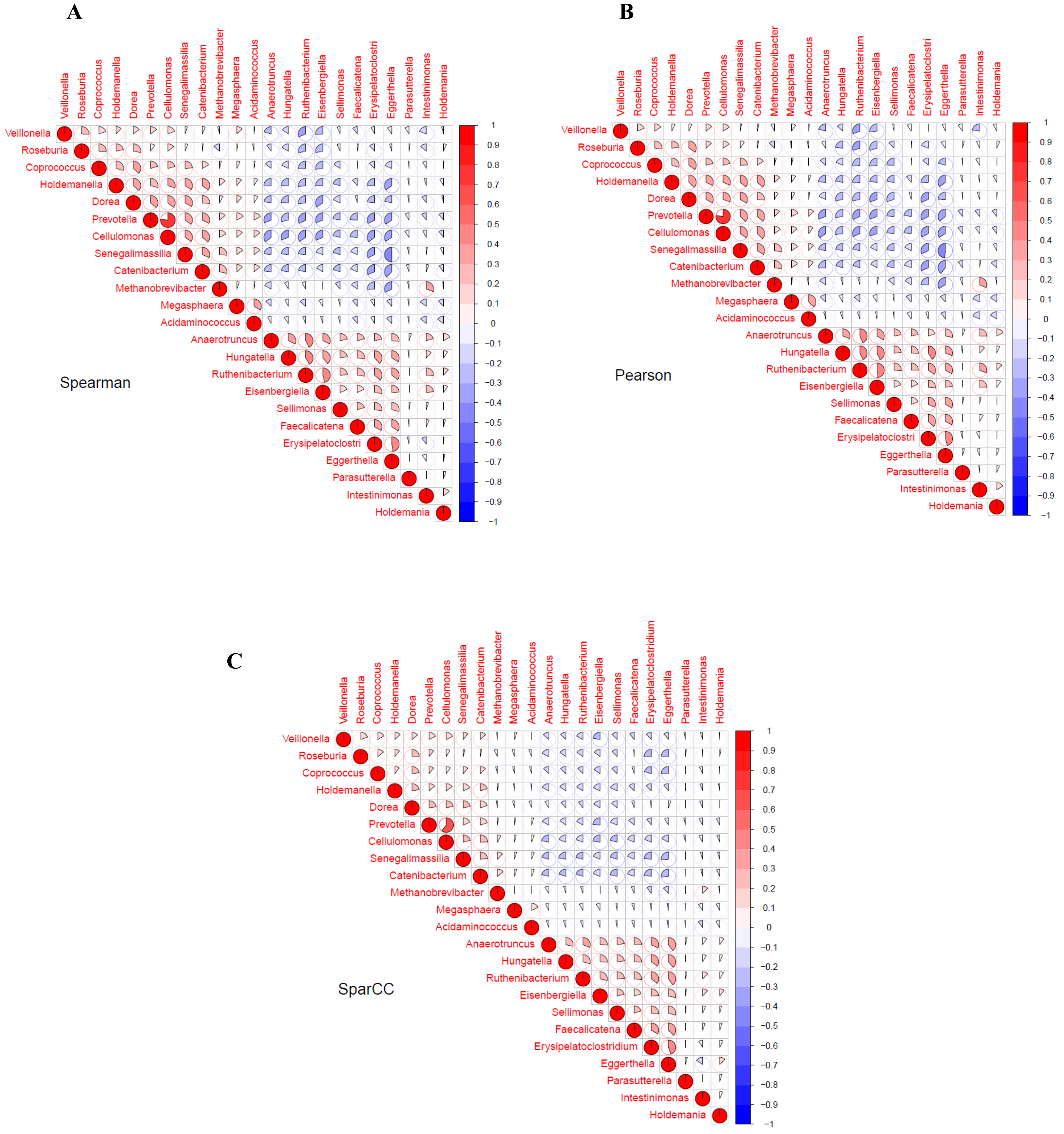

Figure S5. Correlation heatmap for the 23 identified bacterial genera associated with both obesity and US exposure. A. Spearman correlation. B. Pearson correlation. C. SparCC correlation

D.

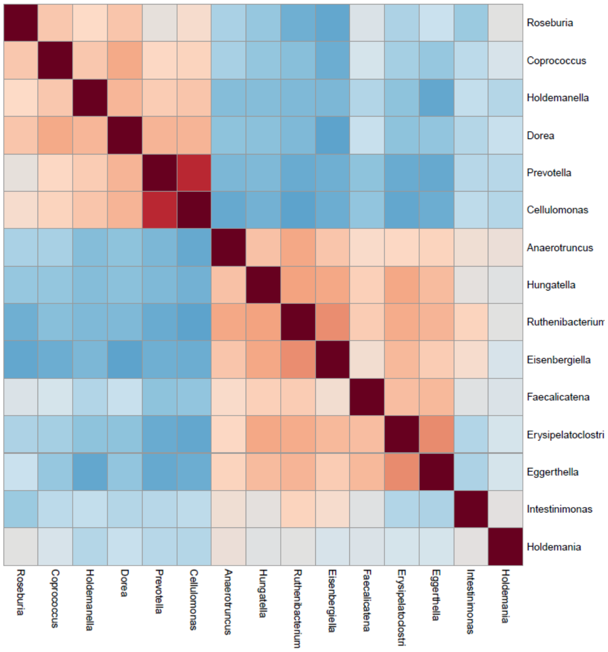

E.

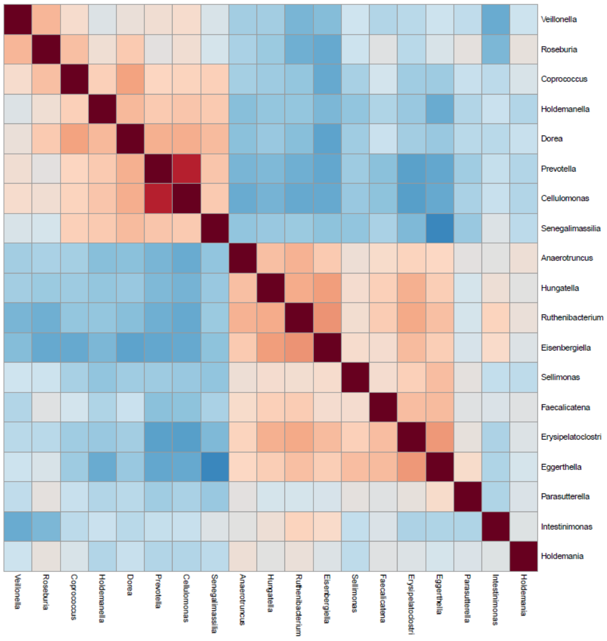

F.

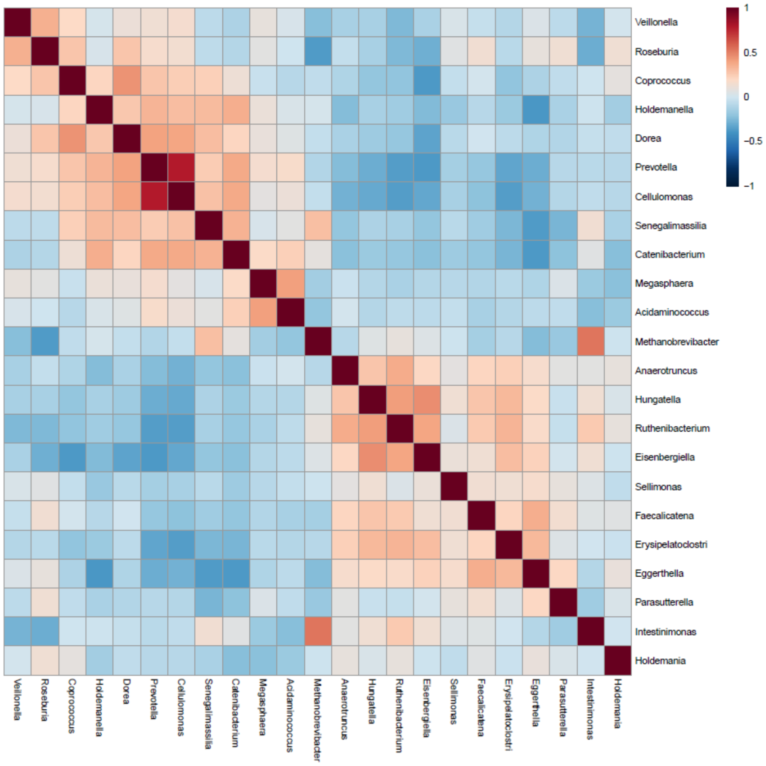

Figure S5. Correlation heatmap for the 23 identified bacterial genera associated with both obesity and US exposure.

D. Sensitivity Analysis included the samples harboring all of the 15 most prevalent genera (n=2220). The prevalence of the 15 key genera >= 90% in the whole study population.

E. Sensitivity Analysis included the samples harboring all of the 19 most prevalent key genera (n=1016). The prevalence of the 19 key genera >= 80% in the whole study population.

F. Sensitivity Analysis included the samples harboring all of the 23 genera (n= 323).

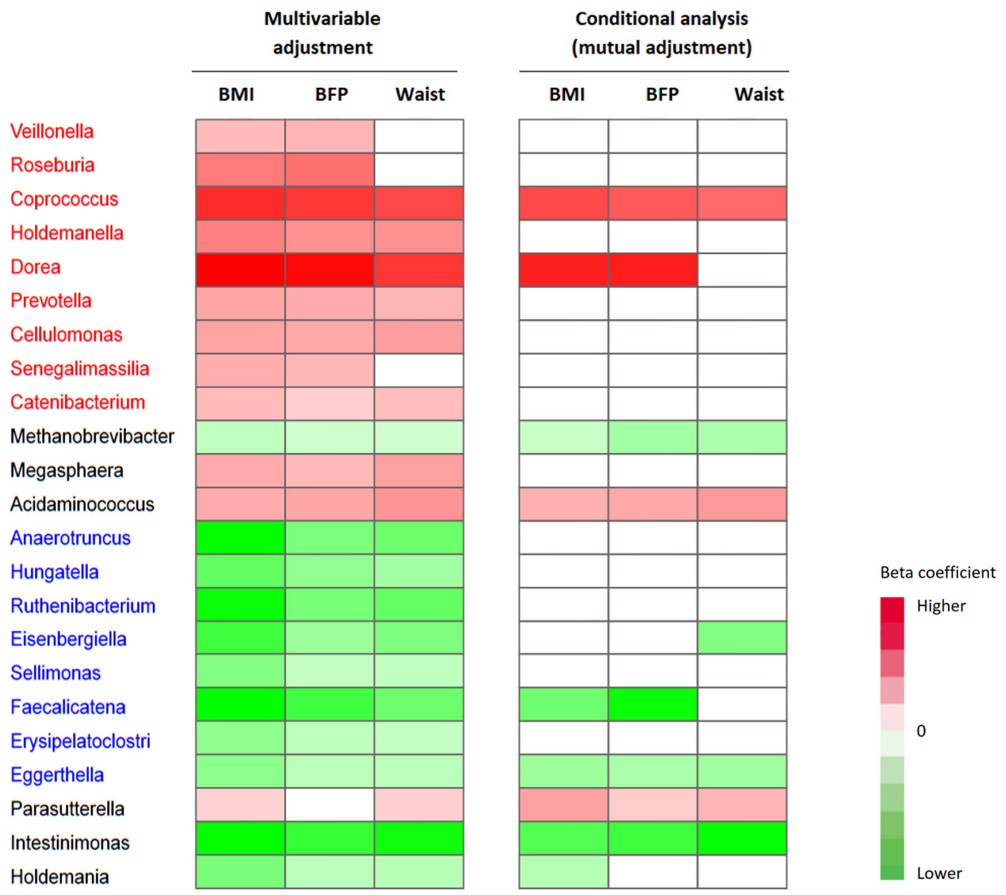

Figure S6. Associations of 23 gut microbial genera with BMI, Body fat percentage and waist circumference. These associations were estimated after adjustment for demographic, socioeconomic, behavioral and clinical factors (multivariable adjustment) and further adjustment for other bacterial genera (conditional analysis, mutual adjustment). Red/green colors depict significant positive/inverse associations and the gradient colors reflect the rank of effect sizes estimated in linear regression models, while white color indicates non-significant associations.

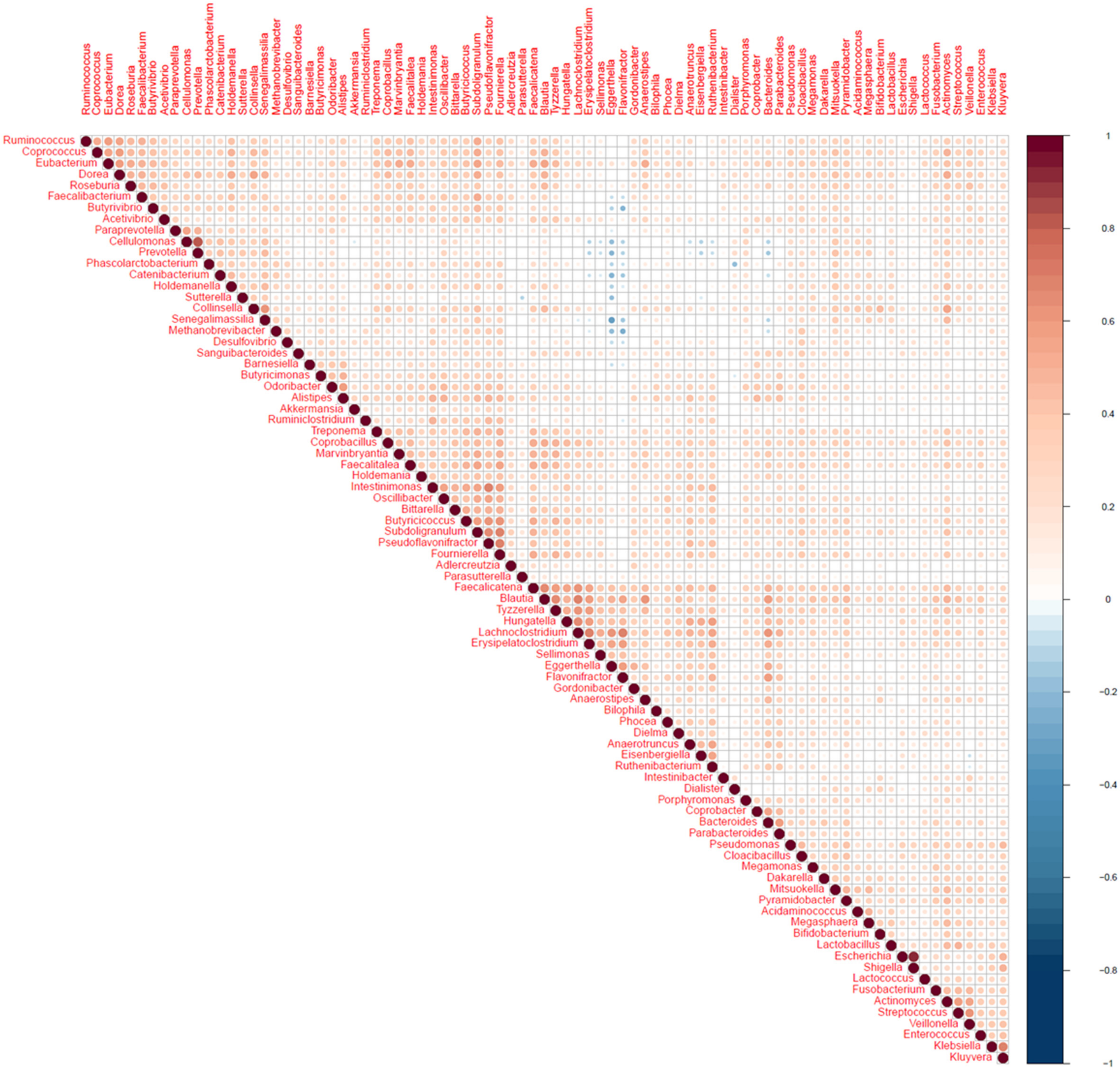

Figure S7. Correlation heatmap for all bacterial genera, using log ratios data and *Clostridium* as reference. The reference genus *Clostridium* was selected based on differential ranking analysis using songbird pipeline, since it was stable (with minimum absolute ranks) across experimental conditions (BMI) and present across most samples. The natural Log-ratio of microbial genera data was calculated using Clostridium as the reference. Significant Spearman correlations ($p < 0.01$) were shown.

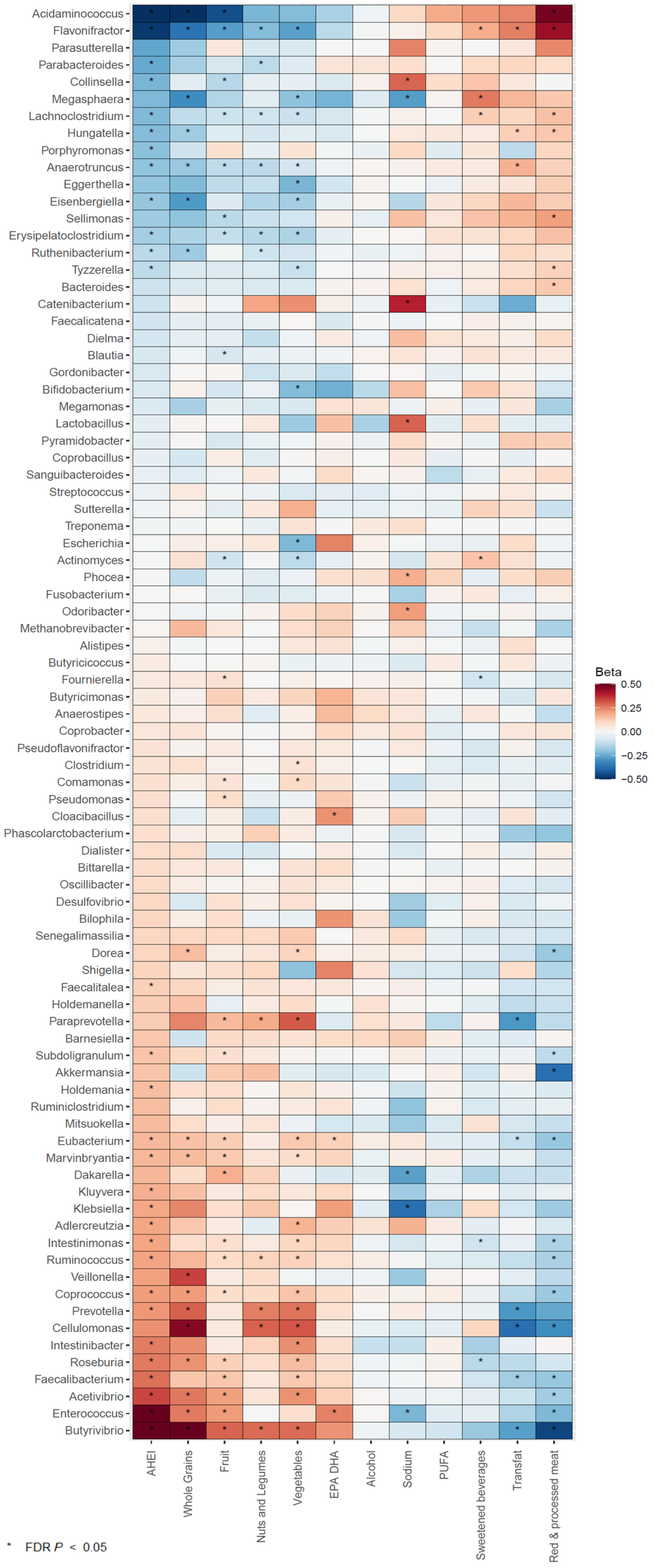

Figure S8. Heatmap for overall dietary quality and individual dietary factor associated with gut microbial genera. Data are effect sizes of dietary factors on gut microbial genera after adjustment for demographic, socioeconomic, behavioral and clinical factors.

Table S1. Characteristics of the study population by US exposure

	Duration of residence in the US (Years)					Mainland US born
	0-15	15-25	25-35	35-45	45+	
No. of participants	455	546	548	413	310	368
Age, year	55.4 (11.2)	55.3 (10.6)	57.5 (8.3)	60.7 (7.5)	65.7 (7.3)	49 (13.2)
Sex, % female	68 %	65%	63%	67%	64%	58%
Hispanic background, %						
Dominican	16%	13%	12%	8%	6%	4%
Central American	8%	12%	16%	9%	3%	1%
Cuban	35%	17%	6%	9%	6%	7%
Mexican	30%	45%	47%	53%	30%	40%
Puerto Rican	3%	3%	11%	17%	51%	45%
South American	9%	10%	6%	4%	4%	2%
Field Center, %						
Bronx	18%	21%	24%	20%	42%	37%
Chicago	15%	30%	30%	37%	26%	25%
Miami	44%	26%	16%	9%	7%	6%
San Diego	20%	21%	28%	32%	24%	30%
Less than high school Education, %	26%	38%	43%	50%	43%	18%
Annually family income, $						
<20,000	47%	45%	40%	46%	52%	34%
20,000-40,000	37%	35%	35%	32%	27%	25%
>40,000	16%	20%	25%	22%	21%	41%
Physical activity, MET-min/day	21.4 (29.6)	22.3 (28.3)	26.9 (45.4)	19.8 (24.1)	21.5 (29.1)	28.9 (36.8)
AHEI score	51.5 (6.8)	52.2 (7.2)	52.6 (7.4)	49.1 (7.3)	49.9 (8.2)	46.2 (7.6)
BMI, kg/m^2	29.6 (5.0)	29.7 (5.1)	30 (5.6)	30.2 (5.7)	30.4 (5.7)	31.1 (7.5)
Waist circumference, cm	98.1(11.7)	99.1(12.5)	100.0(12.7)	100.3(13.3)	101.5(12.9)	102.6(17.4)
Body fat percentage	35.9(8.8)	36.1(8.6)	36.2(8.5)	36.6(8.5)	36.1(8.2)	36.3(10.7)
Host obesity status, %						
Normal	16%	17%	15%	14%	16%	19%
Overweight	42%	40%	39%	42%	38%	31%
Obese I	30%	30%	27%	27%	28%	23%
Obese II	9%	9%	12%	9%	11%	14%
Obese III	3%	4%	6%	7%	6%	11%
Metformin use, %	10%	15%	19%	21%	21%	11%

Table S3. Multivariable regression analyses: 23 genera associated with both obesity and US exposure.

Taxa_Genus	Association with Obesity			Association with US exposure			Direction of association		Expected	Cluster	Association with Fiber		
	Beta	P	P.FDR	Beta	P	P.FDR	Obesity	US exposure			Beta	P	P.FDR
Acidaminococcus	0.024	3.31E-07	4.63E-06	0.321	3.38E-11	2.18E-10	Pos	Pos	Y	-	-0.416	2.55E-05	4.28E-04
Megasphaera	0.024	1.12E-04	5.85E-04	0.090	1.36E-02	2.48E-02	Pos	Pos	Y	-	-0.274	2.24E-04	1.78E-03
Parasutterella	0.013	7.91E-03	2.01E-02	0.148	1.54E-03	3.09E-03	Pos	Pos	Y	-	-0.029	7.63E-01	8.67E-01
Methanobrevibacter	-0.017	2.46E-03	7.66E-03	-0.132	9.24E-04	1.99E-03	Neg	Neg	Y	-	0.048	5.51E-01	7.12E-01
Holdemania	-0.036	3.21E-04	1.35E-03	-0.052	2.30E-02	4.11E-02	Neg	Neg	Y	-	0.094	1.48E-02	4.35E-02
Intestinimonas	-0.099	3.99E-14	3.35E-12	-0.076	1.33E-05	3.60E-05	Neg	Neg	Y	-	0.087	1.40E-02	4.35E-02
Dorea	0.068	2.54E-08	5.61E-07	-0.101	7.62E-08	3.20E-07	Pos	Neg	N	ClusterA	0.093	1.45E-02	4.35E-02
Holdemanella	0.036	5.20E-05	3.12E-04	-0.110	1.68E-05	4.41E-05	Pos	Neg	N	ClusterA	-0.047	3.67E-01	5.05E-01
Roseburia	0.035	1.65E-03	5.34E-03	-0.131	1.15E-10	6.89E-10	Pos	Neg	N	ClusterA	0.167	5.53E-05	6.63E-04
Coprococcus	0.053	2.88E-06	3.22E-05	-0.099	6.10E-07	2.09E-06	Pos	Neg	N	ClusterA	0.108	7.54E-03	2.88E-02
Prevotella	0.021	1.24E-04	6.11E-04	-0.309	2.84E-14	2.65E-13	Pos	Neg	N	ClusterA	0.208	1.25E-02	4.02E-02
Catenibacterium	0.018	1.44E-04	6.73E-04	-0.226	3.65E-06	1.14E-05	Pos	Neg	N	ClusterA	0.045	6.51E-01	7.81E-01
Cellulomonas	0.022	1.99E-04	8.81E-04	-0.374	2.37E-23	1.94E-21	Pos	Neg	N	ClusterA	0.284	2.33E-04	1.78E-03
Senegalimassilia	0.022	3.47E-03	1.04E-02	-0.201	2.11E-11	1.48E-10	Pos	Neg	N	ClusterA	0.095	1.20E-01	2.41E-01
Veillonella	0.018	1.52E-02	3.44E-02	-0.120	1.38E-04	3.30E-04	Pos	Neg	N	ClusterA	0.121	6.10E-02	1.35E-01
Anaerotruncus	-0.062	3.95E-06	3.69E-05	0.162	4.53E-22	1.27E-20	Neg	Pos	N	ClusterB	-0.128	1.99E-04	1.78E-03
Eggerthella	-0.030	3.05E-05	2.33E-04	0.166	2.25E-07	8.22E-07	Neg	Pos	N	ClusterB	-0.128	4.97E-02	1.15E-01
Faecalicatena	-0.075	4.32E-05	2.98E-04	0.058	2.59E-06	8.37E-06	Neg	Pos	N	ClusterB	-0.039	1.19E-01	2.41E-01
Sellimonas	-0.032	6.66E-05	3.73E-04	0.174	1.15E-09	6.44E-09	Neg	Pos	N	ClusterB	-0.146	1.24E-02	4.02E-02
Hungatella	-0.044	4.62E-04	1.85E-03	0.130	8.91E-13	6.81E-12	Neg	Pos	N	ClusterB	-0.107	4.12E-03	1.82E-02
Erysipelatoclostridium	-0.030	3.76E-03	1.09E-02	0.180	4.48E-16	7.53E-15	Neg	Pos	N	ClusterB	-0.104	2.19E-02	6.35E-02
Ruthenibacterium	-0.062	1.71E-07	2.87E-06	0.111	4.68E-09	2.31E-08	Neg	Pos	N	ClusterB	-0.122	1.59E-03	8.90E-03
Eisenbergiella	-0.048	1.41E-09	5.92E-08	0.216	5.55E-14	4.66E-13	Neg	Pos	N	ClusterB	-0.211	3.12E-04	2.02E-03

These associations were estimated after adjustment for demographic, socioeconomic, behavioral and clinical factors.

Table S10. Association of host obesity and US exposure with gut microbiota: species level analyses.

Taxa_Species	Association with Obesity			Association with US exposure			Average Relative Abundance (%)
	Beta	P	P.FDR	Beta	P	P.FDR	
Acidaminococcus_Acidaminococcus_intestini	0.132	9.65E-07	7.19E-05	0.322	1.20E-09	1.32E-08	0.35
Acidaminococcus_Acidaminococcus_fermentans	0.114	4.55E-02	1.81E-01	-0.047	6.28E-02	1.25E-01	0.02
Anaerotruncus_Anaerotruncus_colihominis	-0.346	1.10E-06	7.28E-05	0.162	5.44E-16	1.62E-14	0.12
Anaerotruncus_Anaerotruncus_sp._G32012	-0.315	1.02E-04	2.43E-03	-0.030	9.17E-02	1.69E-01	0.01
Catenibacterium_Catenibacterium_mitsuokai	0.081	2.48E-03	2.74E-02	-0.235	9.99E-06	5.13E-05	0.32
Cellulomonas_Cellulomonas_carbonis	0.096	5.57E-03	4.85E-02	-0.413	7.73E-24	2.88E-21	0.25
Cellulomonas_Cellulomonas_flavigena	0.235	3.38E-02	1.54E-01	-0.004	7.59E-01	8.45E-01	0.00
Coprococcus_Coprococcus_comes	0.181	4.40E-03	4.10E-02	-0.114	4.71E-07	3.19E-06	0.54
Coprococcus_Coprococcus_eutactus	0.050	2.38E-01	4.67E-01	-0.326	1.13E-22	1.68E-20	0.38
Coprococcus_Coprococcus_sp._HPP0048	0.060	2.09E-01	4.39E-01	0.170	1.27E-08	1.15E-07	0.06
Dorea_Dorea_formicigenerans	0.138	4.05E-02	1.74E-01	-0.157	9.55E-14	2.03E-12	0.42
Dorea_Dorea_longicatena	0.265	5.15E-07	4.38E-05	-0.160	2.77E-09	2.75E-08	0.65
Dorea_Dorea_sp._52	-0.147	9.08E-02	2.68E-01	0.005	7.63E-01	8.45E-01	0.01
Eggerthella_Eggerthella_lenta	-0.186	8.39E-06	3.57E-04	0.161	2.33E-06	1.34E-05	0.09
Eggerthella_Eggerthella_sp._YY7918	-0.348	1.16E-04	2.56E-03	0.000	9.98E-01	9.98E-01	0.00
Eisenbergiella_Eisenbergiella_tayi	-0.288	1.24E-10	7.39E-08	0.182	1.01E-08	9.55E-08	0.11
Erysipelatoclostridium_Clostridium_innocuum	-0.196	3.24E-03	3.27E-02	0.114	9.67E-08	7.58E-07	0.08
Erysipelatoclostridium_Clostridium_saccharogumia	-0.081	2.21E-01	4.50E-01	0.036	9.53E-02	1.74E-01	0.01
Erysipelatoclostridium_Clostridium_spiroforme	-0.095	2.92E-02	1.38E-01	0.204	5.99E-10	7.14E-09	0.01
Erysipelatoclostridium_Erysipelatoclostridium_ramosum	-0.097	6.23E-03	5.27E-02	0.212	1.72E-07	1.27E-06	0.09
Faecalicatena_Faecalicatena_contorta	-0.523	1.80E-06	9.75E-05	0.037	4.18E-03	1.19E-02	0.04
Faecalicatena_Faecalicatena_fissicatena	-0.316	4.81E-04	7.97E-03	0.069	1.10E-05	5.56E-05	0.00
Holdemanella_Holdemanella_biformis	0.130	1.05E-02	6.93E-02	-0.148	1.48E-07	1.13E-06	0.17
Holdemania_Holdemania_massiliensis	-0.186	8.41E-04	1.19E-02	-0.067	8.80E-03	2.25E-02	0.02
Hungatella_Hungatella_hathewayi	-0.303	1.94E-05	6.80E-04	0.121	1.55E-09	1.57E-08	0.13
Intestinimonas_Intestinimonas_butyriciproducens	-0.386	1.72E-09	5.13E-07	-0.113	3.94E-07	2.70E-06	0.10
Intestinimonas_Intestinimonas_massiliensis	-0.326	4.96E-07	4.38E-05	-0.086	9.20E-05	3.92E-04	0.07
Megasphaera_Megasphaera_cerevisiae	0.091	2.56E-01	4.88E-01	-0.032	7.09E-02	1.37E-01	0.00
Megasphaera_Megasphaera_elsdenii	0.122	2.93E-03	3.07E-02	-0.030	3.93E-01	5.27E-01	0.04
Megasphaera_Megasphaera_massiliensis	0.141	4.71E-03	4.32E-02	0.216	3.66E-14	8.39E-13	0.03
Megasphaera_Megasphaera_micronuciformis	0.236	9.13E-03	6.53E-02	0.004	7.93E-01	8.60E-01	0.00
Megasphaera_Megasphaera_sp._BV3C161	0.093	5.35E-01	7.30E-01	0.012	2.08E-01	3.27E-01	0.00
Megasphaera_Megasphaera_sp._UPII_135E	-0.281	1.15E-02	7.40E-02	-0.067	1.53E-07	1.15E-06	0.00
Methanobrevibacter_Methanobrevibacter_oralis	-0.113	8.27E-01	9.09E-01	-0.001	6.10E-01	7.21E-01	0.00
Methanobrevibacter_Methanobrevibacter_smithii	-0.106	7.35E-04	1.10E-02	-0.121	7.88E-03	2.03E-02	0.08
Parasutterella_Parasutterella_excrementihominis	0.064	2.45E-02	1.23E-01	0.119	1.84E-02	4.34E-02	0.21
Prevotella_Prevotella_albensis	0.123	5.45E-03	4.85E-02	-0.317	3.59E-23	7.13E-21	0.01
Prevotella_Prevotella_amnii	-0.040	5.80E-01	7.52E-01	-0.068	5.75E-04	2.09E-03	0.00
Prevotella_Prevotella_aurantiaca	-0.182	1.45E-01	3.47E-01	-0.031	7.02E-03	1.87E-02	0.00
Prevotella_Prevotella_baroniae	0.140	3.69E-01	5.92E-01	0.006	5.25E-01	6.50E-01	0.00
Prevotella_Prevotella_bergensis	0.102	1.52E-02	8.88E-02	-0.265	6.77E-15	1.75E-13	0.02
Prevotella_Prevotella_bivia	0.061	9.90E-02	2.77E-01	-0.369	5.97E-22	5.93E-20	0.09
Prevotella_Prevotella_bryantii	0.086	1.91E-02	1.06E-01	-0.389	9.65E-24	2.88E-21	0.02

Prevotella_Prevotella_buccae	0.086	4.34E-01	6.47E-01	-0.033	1.01E-02	2.53E-02	0.01
Prevotella_Prevotella_buccalis	0.019	7.17E-01	8.30E-01	-0.045	9.81E-02	1.78E-01	0.03
Prevotella_Prevotella_copri	0.069	1.66E-03	2.02E-02	-0.532	3.13E-16	1.04E-14	10.99
Prevotella_Prevotella_corporis	0.131	2.86E-03	3.04E-02	-0.099	2.48E-03	7.69E-03	0.01
Prevotella_Prevotella_dentalis	0.082	1.78E-01	3.98E-01	-0.116	7.21E-07	4.67E-06	0.00
Prevotella_Prevotella_dentasini	-0.243	4.63E-02	1.83E-01	-0.061	1.80E-07	1.31E-06	0.00
Prevotella_Prevotella_denticola	0.080	5.62E-01	7.46E-01	-0.043	3.04E-05	1.43E-04	0.00
Prevotella_Prevotella_disiens	-0.017	6.93E-01	8.20E-01	-0.229	1.56E-11	2.27E-10	0.02
Prevotella_Prevotella_enoeca	0.366	1.33E-02	8.00E-02	-0.042	1.21E-05	6.06E-05	0.00
Prevotella_Prevotella_fusca	0.216	2.03E-02	1.07E-01	-0.079	2.43E-07	1.72E-06	0.00
Prevotella_Prevotella_histicola	0.191	4.14E-02	1.75E-01	-0.072	2.49E-06	1.41E-05	0.00
Prevotella_Prevotella_intermedia	-0.169	2.95E-01	5.28E-01	-0.020	2.54E-02	5.71E-02	0.00
Prevotella_Prevotella_maculosa	-0.140	3.00E-01	5.28E-01	-0.016	1.30E-01	2.21E-01	0.00
Prevotella_Prevotella_marshii	0.146	1.07E-01	2.87E-01	-0.049	1.94E-03	6.14E-03	0.00
Prevotella_Prevotella_melaninogenica	0.084	3.13E-01	5.39E-01	-0.100	7.01E-09	6.74E-08	0.00
Prevotella_Prevotella_micans	0.334	1.15E-01	3.01E-01	-0.020	3.63E-03	1.05E-02	0.00
Prevotella_Prevotella_multiformis	0.177	8.51E-03	6.34E-02	-0.149	1.86E-12	3.08E-11	0.00
Prevotella_Prevotella_multisaccharivorax	0.201	1.18E-03	1.56E-02	-0.208	1.15E-19	7.62E-18	0.00
Prevotella_Prevotella_nanceiensis	0.095	3.98E-02	1.72E-01	0.217	2.05E-12	3.30E-11	0.01
Prevotella_Prevotella_nigrescens	-0.013	9.45E-01	9.74E-01	0.002	8.20E-01	8.79E-01	0.00
Prevotella_Prevotella_oris	0.073	7.20E-01	8.30E-01	-0.008	2.44E-01	3.71E-01	0.00
Prevotella_Prevotella_oryzae	0.335	5.32E-02	1.98E-01	-0.047	1.11E-08	1.02E-07	0.00
Prevotella_Prevotella_oulorum	0.080	1.80E-01	4.01E-01	-0.170	1.46E-12	2.49E-11	0.00
Prevotella_Prevotella_pallens	-0.010	8.45E-01	9.12E-01	-0.133	1.57E-06	9.65E-06	0.01
Prevotella_Prevotella_saccharolytica	0.085	6.51E-01	7.93E-01	-0.020	9.28E-03	2.34E-02	0.00
Prevotella_Prevotella_salivae	0.088	4.16E-01	6.34E-01	-0.096	4.31E-13	7.78E-12	0.00
Prevotella_Prevotella_scopos	0.141	1.84E-01	4.07E-01	-0.075	2.98E-08	2.54E-07	0.00
Prevotella_Prevotella_sp._109	0.126	6.56E-02	2.18E-01	-0.012	5.69E-01	6.88E-01	0.18
Prevotella_Prevotella_sp._AGR2160	0.173	9.72E-03	6.72E-02	-0.106	6.86E-07	4.49E-06	0.00
Prevotella_Prevotella_sp._HUN102	-0.072	2.98E-01	5.28E-01	-0.074	3.70E-04	1.43E-03	0.00
Prevotella_Prevotella_sp._P476	0.052	1.58E-01	3.72E-01	-0.312	1.23E-15	3.33E-14	0.19
Prevotella_Prevotella_sp._S7_MS_2	-0.082	2.33E-01	4.62E-01	-0.086	3.52E-05	1.64E-04	0.00
Prevotella_Prevotella_sp._S718	0.059	1.20E-01	3.07E-01	-0.314	9.59E-17	3.57E-15	0.03
Prevotella_Prevotella_stercorea	0.059	6.96E-02	2.25E-01	-0.399	5.22E-20	3.89E-18	0.78
Prevotella_Prevotella_timonensis	-0.050	3.37E-01	5.57E-01	-0.152	3.82E-08	3.21E-07	0.01
Roseburia_Roseburia_faecis	0.168	2.01E-04	3.99E-03	-0.163	2.68E-07	1.87E-06	1.34
Roseburia_Roseburia_hominis	0.078	2.55E-01	4.88E-01	-0.070	8.52E-04	3.00E-03	0.39
Roseburia_Roseburia_intestinalis	0.085	8.13E-02	2.52E-01	-0.204	2.35E-12	3.69E-11	1.54
Roseburia_Roseburia_inulinivorans	0.053	3.75E-01	5.99E-01	-0.197	1.38E-16	4.84E-15	1.82
Ruthenibacterium_Ruthenibacterium_lactatiformans	-0.361	5.83E-08	6.95E-06	0.087	5.39E-05	2.40E-04	0.36
Sellimonas_Sellimonas_intestinalis	-0.184	7.45E-05	2.02E-03	0.144	2.96E-06	1.65E-05	0.03
Senegalimassilia_Senegalimassilia_anaerobia	0.083	5.03E-02	1.92E-01	-0.248	1.81E-13	3.60E-12	0.03
Veillonella_Veillonella_magna	0.225	3.24E-01	5.44E-01	-0.001	8.70E-01	9.15E-01	0.00
Veillonella_Veillonella_parvula	0.070	2.54E-03	8.29E-02	-0.168	1.86E-06	1.11E-05	0.04
Veillonella_Veillonella_seminalis	0.042	6.05E-01	7.62E-01	-0.036	3.72E-02	7.98E-02	0.01

These associations were estimated after adjustment for demographic, socioeconomic, behavioral and clinical factors. FDR *P-values* were calculated based on all the 596 species belong to 84 genera included in the current analysis.

Table S11. Conditional analysis for genera associated with both obesity and US exposure.

Taxa_Genus	Conditional Analysis Obesity Model1		Conditional Analysis US exposure Model1		Conditional Analysis Obesity Model2		Conditional Analysis US exposure Model2		Expected	Cluster
	Beta	P	Beta	P	Beta	P	Beta	P		
Acidaminococcus	0.014	0.004	0.046	0.000	NA		NA		Y	-
Megasphaera	0.004	0.514	0.016	0.133	NA		NA		Y	-
Parasutterella	0.015	0.002	0.020	0.009	NA		NA		Y	-
Methanobrevibacter	-0.019	0.005	0.011	0.285	NA		NA		Y	-
Holdemania	-0.020	0.044	-0.035	0.028	NA		NA		Y	-
Intestinimonas	-0.063	0.000	-0.145	0.000	NA		NA		Y	-
Dorea	0.041	0.004	0.018	0.438	NA		0.009	0.696	Y	ClusterA
Holdemanella	0.007	0.474	0.017	0.279	0.064	0.262	0.016	0.307	Y	ClusterA
Roseburia	0.005	0.667	-0.063	0.002	0.076	0.268	NA		Y	ClusterA
Coprococcus	0.027	0.032	-0.031	0.124	NA		-0.026	0.174	Y	ClusterA
Prevotella	-0.003	0.690	-0.031	0.003	0.029	0.422	NA		Y	ClusterA
Catenibacterium	0.008	0.143	-0.002	0.785	0.034	0.246	0.001	0.867	Y	ClusterA
Senegalimassilia	-0.005	0.551	-0.048	0.001	-0.031	0.545	NA		Y	ClusterA
Veillonella	-0.003	0.704	-0.021	0.086	0.058	0.164	-0.010	0.415	Y	ClusterA
Anaerotruncus	-0.013	0.424	0.182	0.000	-0.089	0.290	NA		Y	ClusterB
Eggerthella	-0.018	0.046	-0.024	0.108	NA		-0.021	0.141	Y	ClusterB
Faecalicatena	-0.050	0.014	0.017	0.214	NA		0.027	0.406	Y	ClusterB
Sellimonas	-0.010	0.272	0.022	0.114	-0.050	0.320	0.023	0.088	Y	ClusterB
Hungatella	0.015	0.304	0.033	0.158	-0.054	0.489	0.020	0.400	Y	ClusterB
Erysipelatoclostridium	0.005	0.680	0.054	0.008	0.057	0.413	NA		Y	ClusterB
Ruthenibacterium	0.009	0.558	-0.024	0.329	-0.092	0.230	0.026	0.274	Y	ClusterB
Eisenbergiella	-0.019	0.064	0.040	0.007	-0.053	0.232	NA		Y	ClusterB

The conditional analysis Model 1 included 22 genera associated with both obesity and US exposure (*Cellulomonas* was excluded due to its high correlation with *Prevotella* and its low relative abundance) in addition to demographic, socioeconomic, behavioral and clinical covariates.

The conditional analysis Model 2 for obesity included four independent BMI-associated genera (*Dorea, Coprococcus, Eggerthella, Faecalicatena*), in addition to demographic, socioeconomic, behavioral and clinical covariates.

The conditional analysis Model 2 for US exposure included six independent US exposure-associated genera (*Anaerotruncus, Eisenbergiella , Erysipelatoclostridium , Roseburia , Prevotella , Senegalimassilia*), in addition to demographic, socioeconomic, behavioral and clinical covariates.

Table S12. Levels of gut microbial genera according to host obesity.

	normal		overweight		obese I		obese II		obese III		*Ptrend*
	lsmean	SE	lsmean	SE	lsmean	SE	lsmean	SE	lsmean	SE	
**Dorea**	9.88	0.07	10.05	0.05	10.13	0.06	10.08	0.08	10.20	0.10	0.004
**Coprococcus**	7.23	0.85	7.39	0.85	7.39	0.85	7.42	0.85	7.58	0.85	0.018
Roseburia	12.30	0.08	12.50	0.06	12.40	0.07	12.40	0.09	12.40	0.12	0.722
Prevotella	11.70	0.15	11.70	0.11	11.50	0.13	11.80	0.19	11.60	0.23	0.552
**Intestinimonas**	7.06	0.06	7.05	0.05	6.99	0.05	6.87	0.08	6.66	0.10	<0.001
**Holdemania**	4.03	0.10	3.86	0.07	3.89	0.08	3.95	0.12	3.55	0.15	0.049
Senegalimassilia	3.89	0.11	3.72	0.08	3.73	0.09	3.79	0.13	3.57	0.17	0.244
Eisenbergiella	4.57	0.10	4.39	0.08	4.40	0.09	4.41	0.13	4.31	0.16	0.188
**Eggerthella**	5.59	0.11	5.47	0.08	5.40	0.09	5.43	0.13	5.11	0.16	0.020
Erysipelatoclostridium	6.88	0.08	6.87	0.06	6.87	0.06	6.88	0.09	6.87	0.12	0.953
Faecalicatena	5.82	0.05	5.72	0.03	5.69	0.04	5.76	0.06	5.62	0.07	0.027
**Anaerotruncus**	6.86	0.06	6.82	0.05	6.82	0.05	6.77	0.08	6.80	0.09	0.401
**Acidaminococcus**	3.93	0.19	3.87	0.14	4.09	0.16	4.29	0.24	4.84	0.30	0.003
**Parasutterella**	4.19	0.20	4.39	0.15	4.69	0.17	4.42	0.24	5.35	0.31	0.003
**Methanobrevibacter**	2.69	0.15	2.78	0.11	2.38	0.13	2.50	0.18	2.19	0.23	0.006

Data are adjusted mean (SE) of each gut microbial genus (CSS transformed relative abundance) across host obesity status after adjustment for demographic, socioeconomic, behavioral and clinical covariates, and other genera (mutual adjustment, conditional analysis). Bold font indicates the microbial genera significantly associated with host obesity status (*Ptrend* < 0.05).

Table S13. Target US exposure associated enzyme selection: Enrichment test for the annotated enzymes

Enzyme LV2	Sig._Enzymes at ELV2 (FDR0.1)	not_sig	Total Enzymes of the categroy (>=10)	P
3.2 Glycosylases	21	52	73	0.001*
2.5 Transferring alkyl or aryl groups, other than methyl groups	12	27	39	0.006
2.3 Acyltransferases	15	65	80	0.151
3.5 Acting on carbon-nitrogen bonds, other than peptide bonds	14	63	77	0.190
3.6 Acting on acid anhydrides	17	80	97	0.201
2.1 Transferring one-carbon groups	17	83	100	0.238
2.4 Glycosyltransferases	16	83	99	0.314
5.4 Intramolecular transferases	7	33	40	0.327
1.6 Acting on NADH or NADPH	5	24	29	0.385
3.4 Acting on peptide bonds (peptidases)	10	53	63	0.390
4.2 Carbon-oxygen lyases	12	66	78	0.414
6.1 Forming carbon-oxygen bonds	5	25	30	0.415
1.8 Acting on a sulfur group of donors	3	17	20	0.546
4.1 Carbon-carbon lyases	12	74	86	0.553
1.3 Acting on the CH-CH group of donors	5	30	35	0.555
4.3 Carbon-nitrogen lyases	2	11	13	0.562
5.1 Racemases and epimerases	3	20	23	0.643
3.1 Acting on ester bonds	14	94	108	0.661
2.6 Transferring nitrogenous groups	5	36	41	0.697
6.3 Forming carbon-nitrogen bonds	6	43	49	0.699
1.17 Acting on CH or CH2 groups	1	9	10	0.779
2.7 Transferring phosphorus-containing groups	35	247	282	0.789
1.4 Acting on the CH-NH2 group of donors	2	19	21	0.813
2.8 Transferring sulfur-containing groups	2	19	21	0.813
1.5 Acting on the CH-NH group of donors	1	13	14	0.879
5.2 cis-trans-Isomerases	1	13	14	0.879
1.1 Acting on the CH-OH group of donors	11	102	113	0.927
1.2 Acting on the aldehyde or oxo group of donors	4	49	53	0.949
1.- other	1	23	24	0.973
1.14 Acting on paired donors, with O2 as oxidant and incorporatio	1	24	25	0.977

*: FDR $P < 0.05$

Table S14. Regression analyses: Enrichment test selected key enzymes associated with both US exposure and fiber intake.

KO group	Association with US exposure			Association with Diet Fiber			Expected	Enzyme Annotations LV4	Enzyme Annotations LV3	Enzyme AnnotationsLV2	Enzyme Annotations LV1
	Beta	P	P.FDR	Beta	P	P.FDR					
K01191	4.46E-04	5.56E-03	0.064	-2.07E-04	3.69E-06	0.000	Y	3.2.1.24 alpha-mannosidase	3.2.1 Glycosidases, i.e. enzymes that hydrolyse O- and S-glycosyl compounds	3.2 Glycosylases	3. Hydrolases
K01250	4.55E-04	4.56E-03	0.061	-2.09E-04	3.11E-06	0.000	Y	3.2.-.-	3.2.- Glycosylases	3.2 Glycosylases	3. Hydrolases
K10563	4.94E-04	7.43E-03	0.072	-2.43E-04	2.31E-06	0.000	Y	3.2.2.23 DNA-formamidopyrimidine glycosylase	3.2.2 Hydrolysing N-glycosyl compounds	3.2 Glycosylases	3. Hydrolases
K01209	-1.44E-03	8.63E-03	0.076	6.04E-04	7.75E-05	0.003	Y	3.2.1.55 alpha-N-arabinofuranosidase	3.2.1 Glycosidases, i.e. enzymes that hydrolyse O- and S-glycosyl compounds	3.2 Glycosylases	3. Hydrolases
K01206	1.71E-03	1.78E-03	0.041	-5.81E-04	1.38E-04	0.004	Y	3.2.1.51 alpha-L-fucosidase	3.2.1 Glycosidases, i.e. enzymes that hydrolyse O- and S-glycosyl compounds	3.2 Glycosylases	3. Hydrolases
K01243	-6.27E-04	8.36E-03	0.074	2.28E-04	5.84E-04	0.011	Y	3.2.2.9 adenosylhomocysteine nucleosidase	3.2.2 Hydrolysing N-glycosyl compounds	3.2 Glycosylases	3. Hydrolases
K01200	1.28E-03	6.19E-03	0.067	-4.41E-04	6.55E-04	0.013	Y	3.2.1.41 pullulanase	3.2.1 Glycosidases, i.e. enzymes that hydrolyse O- and S-glycosyl compounds	3.2 Glycosylases	3. Hydrolases
K01223	-8.94E-04	3.38E-03	0.056	2.57E-04	2.47E-03	0.029	Y	3.2.1.86 6-phospho-beta-glucosidase	3.2.1 Glycosidases, i.e. enzymes that hydrolyse O- and S-glycosyl compounds	3.2 Glycosylases	3. Hydrolases
K01208	-1.62E-03	7.53E-03	0.072	5.00E-04	3.02E-03	0.033	Y	3.2.1.54 cyclomaltodextrinase	3.2.1 Glycosidases, i.e. enzymes that hydrolyse O- and S-glycosyl compounds	3.2 Glycosylases	3. Hydrolases
K12373	4.15E-03	1.32E-02	0.096	-1.36E-03	3.46E-03	0.037	Y	3.2.1.52 beta-N-acetylhexosaminidase	3.2.1 Glycosidases, i.e. enzymes that hydrolyse O- and S-glycosyl compounds	3.2 Glycosylases	3. Hydrolases
K15531	-7.93E-04	9.77E-03	0.083	2.47E-04	3.93E-03	0.040	Y	3.2.1.156 oligosaccharide reducing-end xylanase	3.2.1 Glycosidases, i.e. enzymes that hydrolyse O- and S-glycosyl compounds	3.2 Glycosylases	3. Hydrolases
K01214	-1.82E-03	2.97E-03	0.053	4.81E-04	4.85E-03	0.047	Y	3.2.1.68 isoamylase	3.2.1 Glycosidases, i.e. enzymes that hydrolyse O- and S-glycosyl compounds	3.2 Glycosylases	3. Hydrolases

These associations were estimated after adjustment for demographic, socioeconomic, behavioral and clinical factors.

Table S15. The presence of key enzymes in specific bacteria: Alignment analysis

Enzyme (ID)	Enzyme Annotation	Enzyme Representive Reference Sequences	Enzyme Representive KO group	Bacteria Cluster	Genus	NCBI Reference genomes of the Genus	Presence	Max Score	Total Score	Query Coverage	E value	Percent indentity
EC:3.2.1.156	oligosaccharide reducing-end xylanase	rexA	K11531	Cluster A	***Prevotella***	***Prevotella ruminicola ATCC 19189***	**Y**	**2449**	**2449**	**100%**	**0**	**100.00%**
				Cluster A		***prevotella copri DSM 18205***	**Y**	**34.6**	**13687**	**97%**	**0.078**	**95.24%**
				Cluster A	***Roseburia***	***Roseburia hominis DSM:16839***	**Y**	**2111**	**2111**	**100%**	**0**	**100.00%**
				Cluster A		***Roseburia intestinalis XB6B4***	**Y**	**2128**	**2128**	**100%**	**0**	**100.00%**
				Cluster A	*Dorea*	*Dorea formicigenerans ATCC 27755*	N	No significant similarity found.				
				Cluster A	*Coprococcus*	*Coprococcus comes ATCC 27758*	N	No significant similarity found.				
