# Supplementary material for: Microbial co-occurrence complicates associations of gut microbiome with US immigration, dietary intake and obesity
Source: Genome Biol. 2021 Dec 10;22:336. doi: 10.1186/s13059-021-02559-w (PMC8665519; doi:10.1186/s13059-021-02559-w)
Supplement: Supplementary file 9 — Additional file 9. Review history. [file 13059_2021_2559_MOESM9_ESM.docx]

Review History

**First round of review**

**Reviewer 1**

**Are you able to assess all statistics in the manuscript, including the appropriateness of statistical tests used?** Yes, and I have assessed the statistics in my report.

**Comments to author:**

- Are the methods appropriate to the aims of the study, are they well described, and are necessary controls included? If not, please specify what is required.
Throughout the paper, there is mention of "adjustment for demographic, socioeconomic, behavioral and clinical factors" which are then listed in the Methods. I am surprised to see that Hispanic background was not included. Supplementary material should include an exploration of these other variables, as they are very likely to contribute to some of the gut microbiome variation seen here. It is also not clear how the effect sizes of the variables of interest (US exposure and obesity) compare to those of the other covariates.
While this is clearly an observational study, comparisons against some form of a control group would have been insightful. This could have been done with existing gut microbiome datasets for Caucasian Americans or subjects in this study with <1 or < 5 years of US exposure.
I have concerns about lack of details about the place of origin - especially because it appears as though the Hispanic background was not even included as a covariate in the regression models. Specifically, it would be incredibly important to know whether subjects with a Mexican background came from a highly urban or highly rural part of Mexico--as it is common knowledge that there is enormous diversity in the population, environment, etc. within one country, which will ultimately impact the starting composition of the gut microbiome of newly arrived individuals (as well as their initial BMI). This is also likely what is contributing to the "paradoxical" associations reported.
In general there is not sufficient detail about the methods. Some details in Methods should go into the figure legends -- at the very least, the statistical tests used and cite the p-values whenever possible.
Some minor questions for the authors:
Figure S2 - Why was the cut off for age grouping = 55?
Line 118 - Were the US-born subjects' parents also US-born?
Why is the duration of residence grouped into exposure groups? The cutoffs seem arbitrary and I would much rather see this as a scatter plot, especially since I presume the models were regressions.

- Are the conclusions adequately supported by the data shown? If not, please explain
I have concerns over the methods used in the paper. Particularly, the selection of the "significant" taxa and doing further analysis with only this subset of taxa.
Figure S1B: The R^2 values are much too low to offer that these variables are explaining any variation in beta diversity - regardless of the p-value. The lack of a visual trend in the PCoAs is also not supportive of the finding. Suggestion is to examine more direct correlations of variables with beta diversity distances.
Figure 3B-C - Having Fiber listed in addition to the other items inflates the heatmap because there is significant correlation between fiber and vegetables, fruits, and whole grains.
It is not clear why the authors chose to limit the diet correlations to only the 15 taxa correlated with obesity or US exposure, especially because of the known role that diet plays in modulating the microbiome. I would have expected to see more work done on exploring overall diet-microbiome associations.

- Are sufficient details provided to allow replication and comparison with related analyses that may have been performed? If not, please specify what is required.
Where are the links to the scripts/code?
Because of the highly correlated nature of all of the different covariates with US exposure in general, I would have liked to see more work done to disentangle the relationships between the covariates and to attempt to explain how the gut microbiome could be modulating the observations.

- Does the work represent a significant advance over previously published studies?
Unfortunately, the paper as-is misses the mark. I think it could be incredibly interesting to perform similar analyses within groups from different Hispanic backgrounds. It may be that the authors could uncover interesting findings that point to the urban-rural gradient in the sources of origin (which I acknowledge the authors may not have data for).

- Is the paper of broad interest to others in the field, or of outstanding interest to a broad audience of biologists?
In general, the topic of the paper is of broad interest to biologists but unfortunately the methods and results fall short.

I found Figures 1 and 2 non-intuitive and overly complicated. For example, what is the purpose of showing phylogenetic relatedness in Figure 1A? And what is the overall takeaway for the co-occurrence network?

**Reviewer 2**

**Are you able to assess all statistics in the manuscript, including the appropriateness of statistical tests used?** No, do not feel adequately qualified to assess the statistics.

**Comments to author:**

This manuscript provides a nice analysis of the associations of gut microbiome with US immigration, dietary intake and obesity. It is well written and the findings are interesting and for the most part well presented. The major problem we have with this manuscript is that we are having a hard time wrapping our head around how microbes can be correlated with each other under "paradoxical" circumstances (e.g. low BMI but high US exposure), but then this effect is removed if all of the correlated microbes are considered in the model. If microbes are correlated with an environmental and a clinical factor, and with each other, associations should remain. Clearly, causality and mechanisms are unclear here, and associations might be caused by an independent factor, but it is not clear to us how association would suddenly disappear. Some of the associations, e.g. those with Prevotella, seem to be quite spurious (e.g. figure 2B). So, is it possible that some associations were not that strong to begin with? We think it is a weakness of this manuscript that the main conclusion does not appear to be robust. At least, the conceptual framework on which the main conclusion rests is not well enough introduced (e.g. from an ecological perspective), discussion of the mechanisms behind this co-occurrence are lacking, and the manuscript would benefit from an inclusion of implications/big-picture perspective of these results (i.e. Why should it be considered in future studies? What does it tell us?).

Specific points:

* Abstract: As written, the abstract is not clear, especially in relation to 'co-occurrence'.
* Page 4, Line 80: Since the main takeaway of this paper is how the concept of microbial co-occurrence may contribute to the associations between immigration and obesity, perhaps you can include that the complex interactions among microbes may also contribute to host health and the inconsistencies observed in human studies.
* Page 4, Line 85: The way this sentence is written, it sounds as if the immigrants were the ones who conducted the study. Suggest removing "immigrants from Thailand to the US found that" and simply starting the sentence with "US immigration from Thailand was associated…".
* Page 5, Line 97: Were there specific countries this study population came from? There isn't any information in the main text that describes what countries the immigrants came from. Since US Hispanics/Latinos is a broad term, it would be useful to know when reading the main text if the participants came from a certain set of countries so that the conclusions are specific those countries.
* Page 7, Line 156: Why stick with describing the associations at the genera level only? Even if one or two major species were driving the effects seen at the genera level, this is still more informative results than just genera level. Having access to species level data from metagenomics seems to be a missed opportunity.
* Page 7, Line 160: "As" is not necessary in this sentence. Alternatively, "As GMB data tend to be zero-inflated, this may influence the correlation estimates".
* Page 8, Line 167: There seems to be a lot of emphasis on these paradoxical patterns. However, are there not individuals in the cohort who remained at a lower BMI even years after immigration? Or, alternatively, are there not immigrants that have a high BMI even before immigrating? Both might explain some of these patterns.
* Page 8, Line 170: Suggest including a definition of what microbial co-occurrence exactly is and what it means in this context. Further, why should studies adjust for microbial co-occurrence? Perhaps include a brief explanation as to why this should happen.
* Page 9, Line 193: What results are being referred to here: "we examined the associations of these genera with two other obesity measures, body fat percentage and waist circumference, and severity of obesity, and the results were consistent". In other words, what was the consistent result? Suggest defining this so that it is clearer for the reader. Also, how is "severity of obesity" defined or what does it refer to exactly? BMI classification? Is this not already assessed in the initial analysis?
* Page 9, Line 199: "…also observed significant positive correlations among Coprococcus, Roseburia, Prevotella and Dorea" Does this mean these genera co-occur? It is unclear what this means.
* Page 12, Line 264: In the corresponding figure, it doesn't seem like there was much of a difference among the immigrant groups but only a significant decrease in the US-born group. Can the authors comment on whether there were significant differences among immigrant groups?
* Page 14: Lines 318-324: The authors write here about strains within the species Prevotella, but provide absolutely no analysis of those in this manuscript. It would be good to include a strain analysis if it is discussed. This is especially important as there is a claim here that the "co-occurrence hypothesis" is better than the "multiple strain hypothesis".
* Page 15, Line 327: This last sentence of the paragraph is hard to understand. Are the authors saying that Prevotella does not truly correlate with/influence health but rather it is the taxa that correlate with it instead?
* Page 17, Line 371: Were participants excluded if they used antibiotics/probiotics, or was this simply recorded in the study? If it is the former, please include this information. If it is the latter, how was this controlled for in the study?
* Figures (overall): If possible, perhaps the figures with the red and green could be changed to red and blue (e.g. Figure 1A, 2B, 3D, 4B). The red and green might be difficult to differentiate for people with colorblindness.
* Figure 1A: Can something be added to this figure to show which ring represents BMI and which represents US immigration? It's a bit confusing until you read the figure caption, so if something can be added to the figure this would aid in understanding.
* Figure 2A: Please include a label for the two clusters and which one refers to BMI and which one refers to US immigration.
* Figure 2B: At first, it wasn't obvious that the taxa listed in Figure 2A also were aligned with what was being depicted in Figure 2B. It would improve understanding if the colours aligned as well (i.e. the red taxa align with the red boxes indicating those taxa associate with BMI; perhaps use dark blue in Figure 2B instead of green).

**Point-by-point responses to the reviewers’ comments:**

Please find below point-by-point response to the comments. All changes to the manuscript are marked in the text.


***Reviewer #1:***

*- Are the methods appropriate to the aims of the study, are they well described, and are necessary controls included? If not, please specify what is required.
Throughout the paper, there is mention of "adjustment for demographic, socioeconomic, behavioral and clinical factors" which are then listed in the Methods. I am surprised to see that Hispanic background was not included.*

***Response 1:*** We thank the reviewer for these very helpful comments. In our previous version, we did not adjust for Hispanic background in the analyses due to potential collinearity issues as Hispanic background is closely related to study centers. As suggested, in the revision, we have further adjusted for “Hispanic background” as a covariate in the analyses, and the results did not change materially (**Table S5**). In addition, we conducted stratified analyses by Hispanic background to further control for potential confounding effects and did not observe significant heterogeneity across Hispanic background groups (**Table S6** and **Table S7**. Please also see **Response 15**). These results suggest that Hispanic background is less likely to confound the observed associations of gut bacterial genera with US exposure and obesity.

*Supplementary material should include an exploration of these other variables, as they are very likely to contribute to some of the gut microbiome variation seen here. It is also not clear how the effect sizes of the variables of interest (US exposure and obesity) compare to those of the other covariates.*

***Response 2:*** we thank for this very helpful suggestion. In the revision, we have added a series of permutational multivariate analysis of variance (PERMANOVA) analyses to explore and to quantify the gut microbiome variance explained by multiple variables, including Socio-demographics (US exposure, age, sex, study center, Hispanic background, education level, income), behavior (physical activity, total energy intake), Disease and medications (obesity, diabetes, antibiotic use, and metformin use). According to the R^2^, US exposure was the top variable associated with GMB and explained more proportion of GMB variance than other variables. Also, obesity was among the top variables associated with GMB, with the largest R^2^ in the “Disease and medications” category. We have shown these results in **Figure S2** in which these variables were ranked by the estimated R^2^.

*While this is clearly an observational study, comparisons against some form of a control group would have been insightful. This could have been done with existing gut microbiome datasets for Caucasian Americans or subjects in this study with <1 or < 5 years of US exposure.*

***Response 3:*** We thank the reviewer for this suggestion. We performed beta diversity Principal-coordinates analysis (PCoA) to compare the overall community level gut microbial compositions of HCHS/SOL GOLD, versus American Gut Project, and UK Gut Project. As shown in the plot below, the Hispanic/Latino population harbors a quite distinct GMB composition as compared with samples in the American Gut Project and UK Gut Project. This difference could be due to genetic background, socio-demographics, diet/lifestyle, behaviors and many other study characteristics, as well as different methods used to collect stool samples and to measure GMB. Thus, samples from other studies might not be comparable. However, we would appreciate reviewer’s further advice to specify which analyses we might need to do using data from other studies.

In one of our major analyses, we examined associations between US exposure and gut bacterial genera in which participants born in the US served as a group with higher US exposure, while participants recently immigrated to the US served as a group with lower US exposure. Unfortunately, our study did not have participant with <5 years of US residence, but used participants with 5-15 years of US residence as our lowest US exposure group. We have acknowledged this limitation in the revised manuscript. However, even though these participants already had 5-15 years of US exposure, we still found significant differences in gut microbiome composition and bacterial genera when comparing this lowest US exposure group with highest US exposure group (US-born participants). We would observe more stronger results if we compare participants with <5 year of US exposure with US-born participants, and thus this limitation is less likely to change our overall conclusion regarding the relationship between US exposure and GMB. In our other major analyses, we examined associations between gut bacterial genera and obesity in the overall samples as well as stratified by US exposure. We did not found significant differences in the associations between gut bacterial genera and obesity across US exposure groups (**Figure S3C**), suggesting that US exposure might not influence the association between gut bacterial genera and obesity.

**Figure.** Distinct GMB compositions among HCHS/SOL GOLD, American Gut Project, and UK Gut Project: beta diversity Principal-coordinates analysis.

*I have concerns about lack of details about the place of origin - especially because it appears as though the Hispanic background was not even included as a covariate in the regression models.* *Specifically, it would be incredibly important to know whether subjects* *with a Mexican background came from a highly urban or highly rural part of Mexico--as it is common knowledge that there is enormous diversity in the population, environment, etc. within one country, which will ultimately impact the starting composition of the gut microbiome of newly arrived individuals (as well as their initial BMI This is also likely what is contributing to the "paradoxical" associations reported. ).*

***Response 4:*** We thank the reviewer for this very helpful comment. In the revised manuscript, we have adjusted for Hispanic background as a covariate in our analyses, and the overall results did not change (please see **Response 1**). As suggested, we looked at the details about the place of origin for participants with Mexican background and found that most of these participants (91%) came from two major cities: Tijuana (n= 77.5%) and Mexicali: (n=13.5%). Thus, we were unable to compare the potential urban-rural differences due to very few participants who came from rural areas. We have acknowledged this as a limitation in the revised manuscript (Line 408).

However, although we could not examine the potential influences of place of origin within one country, we were able to examine the potential influences of place of origin at the country/area level, since our Hispanic immigrants came from 6 countries/areas (Dominica, Cuba, Puerto Rica, Mexico, Central America, and South America). After excluding US-born participants, we examined associations of gut bacterial genera with US exposure and BMI stratified by 6 countries/areas, and did not find significant heterogeneity in associations across 6 countries/areas. These results have been shown in **Table S8 and Table S9** and also described in the Result section (Line 179).

*In general there is not sufficient detail about the methods. Some details in Methods should go into the figure legends --* *at the very least, the statistical tests used and cite the p-values whenever possible.*

***Response 5:*** We thank the reviewer for this very helpful suggestion. We have added details in our Method section as well as in the supplemental material. In addition, we have updated the figure legends in the revised manuscript and included the detailed information.

*Some minor questions for the authors:
Figure S2 - Why was the cut off for age grouping = 55?*

***Response 6:*** In **Figure S2**, considering the statistical power of the analysis, we set the cut off of age group to 55, which is the median age of participants. So we have equal numbers in each age group and get the maximum power. We have added this information in the text (Line 538).

*Line 118 - Were the US-born subjects' parents also US-born?*

***Response 7:*** No. In this study, most (99 %) of the US-born participants’ parents were Latin America-born.

*Why is the duration of residence grouped into exposure groups? The cutoffs seem arbitrary and I would much rather see this as a scatter plot, especially since I presume the models were regressions.*

***Response 8:*** We grouped the duration of residence into ordinal categorical exposure groups because our analysis included the “US born” group, which represented the highest level of US exposure and could not be simply measured using continuous variable “duration of residence (years)”. In other words, we assume that the US exposure level is higher for US-born Hispanics compared to 1^st^-generation immigrant Hispanics with same years of US residence. Thus, the continuous variable cannot be used in our main analysis on the associations between US exposure and gut microbiome features.

**Figure. Distribution of the Duration of residence in US**

For the cutoffs of US exposure groups, we considered both the data distribution (as shown in above plot) and sample size in each group, to get better power in the analysis. Five levels of US exposure groups were defined: <15, n=455; 15-25, n=546; 25-35, n=548; 35-45, n=413; >45, n=310; and US born, n=368.

*- Are the conclusions adequately supported by the data shown? If not, please explain
I have concerns over the methods used in the paper. Particularly, the selection of the "significant" taxa and doing further analysis with only this subset of taxa.*

***Response 9:*** We thank the reviewer for the comments and would like to clarify our analysis strategy in this study. First, we examined associations of all the taxa (84 genera), not the subset of taxa, with US exposure and obesity (two primary analyses of this study). These primary analyses suggested some unexpected paradoxical associations of some taxa with US exposure and obesity, and thus we performed further analyses to explore the potential explanations for these paradoxical associations by focusing on the taxa (23 genera) that were significantly associated with both US exposure and BMI. Results from these further analyses suggested that microbial co-occurrence might help explain the observed paradoxical associations. This is our major finding which provides a new message in the field: microbial co-occurrence could be an important factor to consider in future studies relating individual GMB taxa to environmental factors and host health and disease. However, we also agree with the reviewer that results on other taxa are also important. Thus, in the revised manuscript, we have added a supplemental table (**Table S2**) to show the results on the associations of all taxa (84 genera) with US exposure and BMI. In addition, we also performed analysis to examine the associations of all taxa with host dietary intake, and results were shown in **Figure S8**.

*Figure S1B: The R^2 values are much too low to offer that these variables are explaining any variation in beta diversity - regardless of the p-value. The lack of a visual trend in the PCoAs is also not supportive of the finding. Suggestion is to examine more direct correlations of variables with beta diversity distances.*

***Response 10:*** We thank the reviewer for the comments. We agree with the reviewer that the R^2^ values are low and these variables might only explain a small proportion of variation in beta-diversity. Our results are consistent with those observed in our previous analyses using 16S data (Kaplan et al., Genome Biol. 2019;20:219-019-1831-z.). Our results are also in line with previous findings observed in large population-based studies in which multiple host factors showed significant associations with beta-diversity but had very small R^2^ values (most variables had R^2^ <0.02) (He at al., Nat Med. 2018;24:1532-1535). Ethnicity has been shown to have a relatively large R^2^ (0.05~0.06) but no clear separation was observed in the PCoA plot (Please see a Figure below from Deschasaux et al. Nat Med. 2018;24:1526-1531 as an example). Taken together, previous findings have suggested that multiple host factors are significant associated with beta-diversity but explain small variation in beta-diversity. Therefore, in the current study, our primary aim is not to examine host factors associated with bacterial beta-diversity but focus on individual taxa in relation to US exposure, dietary intake and obesity. Our results on beta-diversity are confirmatory and thus are shown as supplemental data (**Figure S1B**, **Table S1**)

**Figure. Dissimilarities in gut microbiota composition in the multiethnic HELIUS study. (Deschasaux et al. Nat Med. 2018;24:1526-1531)**

*Figure 3B-C - Having Fiber listed in addition to the other items inflates the heatmap because there is significant correlation between fiber and vegetables, fruits, and whole grains.*

***Response 11:*** We thank the reviewer for this helpful suggestion. We have updated the **Figure 3B and 3C** and removed the row/column of Fiber.

*It is not clear why the authors chose to limit the diet correlations to only the 15 taxa correlated with obesity or US exposure, especially because of the known role that diet plays in modulating the microbiome. I would have expected to see more work done on exploring overall diet-microbiome associations.*

***Response 12:*** We thank the reviewer for the comments. As we mentioned earlier, our primary aim is to clarify the unexpected paradoxical associations of individual taxa with US exposure and obesity. Since the diet tended to change after immigration and that can profoundly influence both body habitus and GMB features, we focused on the associations between diet and 15 taxa that were associated with both US exposure and obesity. However, we agree with the reviewer that the overall diet-microbiome associations are also interesting. Thus, in the revised manuscript, we have examined the associations of host dietary intakes and all taxa and results were shown in **Figure S8** and described in the results section (Line 265). Interestingly, some taxa that associated with both US exposure and obesity were also among the top ones associated with host dietary intakes (e.g., Acidaminococcus). This further supports an important role of diet in the relationships among US exposure, gut microbiome and obesity.

*- Are sufficient details provided to allow replication and comparison with related analyses that may have been performed? If not, please specify what is required.
Where are the links to the scripts/code?*

***Response 13:*** We thank the reviewer for this question. We have added links to the scripts/code in the Method section (line 460- 557).

*Because of the highly correlated nature of all of the different covariates with US exposure in general,* *I would have liked to see more work done to disentangle the relationships between the covariates and to attempt to explain how the gut microbiome could be modulating the observations.*

***Response 14:*** we thank the reviewer for the comments and suggestions. We agree with the reviewer that multiple covariates are correlated with US exposure, and thus to disentangle the effects of US exposure and other covariates on bacterial taxa, we conduct multi-variable regression by including US exposure and all other covariates in the same model. After controlling for these demographic, social-economic, behavioral and clinical factors, we found US exposure was significantly associated with a total of 49 taxa, suggesting these observed associations between US exposure and taxa might be independent of these covariates. Among these 49 taxa which were significantly associated with US exposure, a majority of them were not associated with other covariates. In the revised manuscript, we have added a supplemental Table (**Table S4**) to show all the associations of US exposure and other covariates with all taxa.

*- Does the work represent a significant advance over previously published studies?
Unfortunately, the paper as-is misses the mark.* *I think it could be incredibly interesting to perform similar analyses within groups from different Hispanic backgrounds. It may be that the authors could uncover interesting findings that point to the urban-rural gradient in the sources of origin (which I acknowledge the authors may not have data for).*

***Response 15:*** We thank the reviewer for the comments and suggestions. As suggested by the reviewer, we performed the stratified analyses by Hispanic backgrounds. The associations of bacterial genera with BMI and US exposure were examined in 6 strata of “Hispanic background” (i.e. Dominican, Cuban, Puerto Rican, Central American, Mexican, and South American), and generally consistent results were observed across different Hispanic background. These results suggested that the Hispanic background is less likely to influence the observed paradoxical associations. In the revised manuscript, we have shown these results in **Table S6 and S7** and also described these results in the Result section (Line 175-). As also acknowledged by the reviewer*,* we do not have data to compare potential urban-rural differences, and thus has acknowledged this as a limitation in the revised manuscript (Line 408).

We would like to highlight the significant advance over previously published studies. First, we identified several gut bacterial genera associated with US immigration, host dietary intake and obesity in expected directions, suggesting a potential involvement of these bacteria linking US immigration with obesity, potentially relate to westernized diet during acculturation process. These findings may help better understanding the interrelationship among US immigration, dietary acculturation, and obesity through gut microbiome. Another major contribution of our study is that we demonstrated that bacterial correlations/co-occurrence might lead to some unexpected paradoxical associations of gut bacterial taxa with host factors, while after controlling for bacterial correlations/co-occurrence, these paradoxical associations disappeared. Thus, potential microbial co-occurrence may be an important factor to consider in future studies relating individual GMB taxa to environmental factors and host health and disease.

In the revised manuscript, we added several paragraphs in the Discussion section to discuss significant advance, the implications of the results, and the potential meaning for future studies (Line 354- ; Line 376- ; Line385- )

*- Is the paper of broad interest to others in the field, or of outstanding interest to a broad audience of biologists?
In general, the topic of the paper is of broad interest to biologists but unfortunately the methods and results fall short. I found Figures 1 and 2 non-intuitive and overly complicated. For example, what is the purpose of showing phylogenetic relatedness in Figure 1A? And what is the overall takeaway for the co-occurrence network?*

***Response 16:*** We thank the reviewer for the comments and questions, and would like to provide more justifications for the use of these figures. The phylogenetic tree in **Figure 1A** provided important information in terms of biology taxonomical classification and evolution. In general, one bacteria family consists of structurally or phylogenetically related bacteria genera, and the phylogenetically closely related bacteria genera are more likely to appear similar phenotypes or biology features. In our study, some genera from different phylum were associated with both obesity and US exposure and also correlated with each other (e.g., Prevotella in the Bateroidetes Phylum and Dorea in the Firmicutes Phylum). Thus, their relationships could not be simply explained by the genetic similarity, and there could be some more complicated interactions such as potential co-occurrence between bacteria. In the revised manuscript, we have described this in the result section (Line 246).

The phylogenetic tree plot provides information on the genetic relationships among the genera, while the co-occurrence network in **Figure 2B** provides information on the phenotypic relationships among the genera. For example, Coprococcus and Dorea which were independently associated with higher BMI, and Roseburia, Prevotella and Senegalimassilia which were independently associated with less US exposure, clustered together and showed bacterial co-occurrence relationships. This might explain the unexpected paradoxical associations of these 5 genera with higher BMI and less US exposure. The unexpected associations of Roseburia and Prevotella with host obesity might be induced by their co-occurrence with these obesity-associated taxa (i.e., Coprococcus and Dorea), while the unexpected associations of Coprococcus and Dorea with less US exposure might be induced by their co-occurrence with less US exposure-associated taxa (i.e., Roseburia, Prevotella and Senegalimassilia).

***Reviewer #2:***

*This manuscript provides a nice analysis of the associations of gut microbiome with US immigration, dietary intake and obesity. It is well written and the findings are interesting and for the most part well presented.
The major problem we have with this manuscript is that we are having a hard time wrapping our head around how microbes can be correlated with each other under "paradoxical" circumstances (e.g. low BMI but high US exposure), but then this effect is removed if all of the correlated microbes are considered in the model. If microbes are correlated with an environmental and a clinical factor, and with each other, associations should remain.*  *Clearly, causality and mechanisms are unclear here, and associations might be caused by an independent factor, but it is not clear to us how association would suddenly disappear. Some of the associations, e.g. those with Prevotella, seem to be quite spurious (e.g. figure 2B). So, is it possible that some associations were not that strong to begin with?*

***Response 17:*** We thank the reviewer for these constructive comments. We would like to use two gut microbes, microbe A and microbe B, to better describe the paradoxical associations and how these associations can be explained by the correlations between gut microbes. For example, microbe A is truly associated with high US exposure but not truly associated with low BMI, while microbe B is truly associated with low BMI but not truly associated with high US exposure, and these two microbes are correlated with each other. If we only include microbe A in the regression model on BMI, we would observe that microbe A was associated with low BMI (this is the paradox) because of its correlation with microbe B. However, if we include both microbes in the regression model on BMI, the association between microbe A and low BMI would disappear, while the association between microbe B and low BMI would remain, as microbe B is the one truly associated with low BMI. We would also like to use a figure below to demonstrate that abolished associations for some microbes were not due to weak associations. The figure below shows associations of microbes with BMI before and after the mutual adjustment (all 23 microbes in the same model). For example, Prevotella and Acidaminococcus were both positively associated with BMI and the effect sizes were similar before mutual adjustment. After mutual adjustment, the association between Prevotella and BMI disappeared, while the association between Acidaminococcus and BMI remained significant although the effect size was attenuated. Some microbes had weaker associations with BMI compared to Prevotella before mutual adjustment (e.g. Parasutterella), but their associations with BMI remained significant after mutual adjustment. Some microbes had stronger associations with BMI compared to Prevotella before mutual adjustment (e.g., Roseburia), and their associations with BMI also disappeared. These results suggested that the disappeared associations in the mutual adjustment model were not due to weak associations before mutual adjustment.

**Figure. Associations of gut bacteria genera with obesity, before and after mutual adjustment**
“ * ” indicated the microbial genera which were independently associated with obesity after mutual adjustment.

*We think it is a weakness of this manuscript that the main conclusion does not appear to be robust. At least, the conceptual framework on which the main conclusion rests is not well enough introduced (e.g. from an ecological perspective), discussion of the mechanisms behind this co-occurrence are lacking, and the manuscript would benefit from an inclusion of implications/big-picture perspective of these results (i.e. Why should it be considered in future studies? What does it tell us?).*

***Response 18:*** We thank the reviewer for these constructive suggestions. As suggested, we have added more introductions on the conceptual framework in the Background section to better describe the complex interactions among microbes and their implications in the study of gut microbiota and host health in human studies (Line 105-). We have added one paragraph in the Discussion section to discuss the potential mechanisms underlying the interaction and co-occurrence among microbes, the implications and big-picture perspective of the results, and the meaning for future studies (Line 385- ).

*Specific points:

*       Abstract: As written, the abstract is not clear, especially in relation to 'co-occurrence'.*

***Response 19:*** We appreciate for this helpful suggestion. We have revised the abstract by adding more descriptions on “co-occurrence” . (line 60-)

**       Page 4, Line 80: Since the main takeaway of this paper is how the concept of microbial co-occurrence may contribute to the associations between immigration and obesity, perhaps you can include that the complex interactions among microbes may also contribute to host health and the inconsistencies observed in human studies.*

***Response 20:*** We thank the reviewer for this helpful suggestion. We have added more introductions on the complex interactions among microbes and their implications in host health in human studies in the Background section. (Line 105-)

**       Page 4, Line 85: The way this sentence is written, it sounds as if the immigrants were the ones who conducted the study. Suggest removing "immigrants from Thailand to the US found that" and simply starting the sentence with "US immigration from Thailand was associated…".*

***Response 21:*** Thanks a lot. We have rewritten the sentence according to this helpful suggestion.

**      Page 5, Line 97: Were there specific countries this study population came from? There isn't any information in the main text that describes what countries the immigrants came from. Since US Hispanics/Latinos is a broad term, it would be useful to know when reading the main text if the participants came from a certain set of countries so that the conclusions are specific those countries.*

***Response 22:*** Thank you for the suggestion to clarify this important point. In this study, the Hispanic immigrants came from 6 countries/areas: Dominica, Cuba, Puerto Rica, Mexico, Central America, and South America. We have added the descriptions of Hispanic origin in the main text (Line 120; Line 545).

***       *Page 7, Line 156: Why stick with describing the associations at the genera level only? Even if one or two major species were driving the effects seen at the genera level, this is still more informative results than just genera level. Having access to species level data from metagenomics seems to be a missed opportunity.*

***Response 23:*** we thank the reviewer for the helpful comments and suggestions. We totally agree with the reviewer that species level data may provide more information in addition to the genus level data. In the revised manuscript*,* we have performed the taxonomic analyses at both genus level and species level. The taxonomic species level results are shown in **Table S10 and also described in the main text (Line 185).** The specie-level results were generally consistent with the genus level results, as similar paradoxical associations with host obesity and US exposure were observed. To better interpret our results in the mutual adjustment models and the co-occurrence network which can help explain the observed paradoxical associations, we used genus level data rather than species level data as the number of species (>500 species) is too large. It would be very hard to interpret the mutual adjustment models and the co-occurrence network with a large number of species. To simplify the conditional analysis mutual adjustment models, we performed the conditional analysis at genus level, which allowed a more parsimonious explanation of the data with fewer parameters, better statistical power, and a simpler and more informative interpretation of the results.

**       Page 7, Line 160: "As" is not necessary in this sentence. Alternatively, "As GMB data tend to be zero-inflated, this may influence the correlation estimates".*

***Response 24:*** Thanks a lot. We have corrected this sentence accordingly. **       Page 8, Line 167: There seems to be a lot of emphasis on these paradoxical patterns. However, are there not individuals in the cohort who remained at a lower BMI even years after immigration? Or, alternatively, are there not immigrants that have a high BMI even before immigrating? Both might explain some of these patterns.*

***Response 25:*** We thank the reviewer for the comments and questions, and would like to clarify that our analyses on the associations of taxa with host obesity and US immigration were conducted at the population level. It is not uncommon to observe individuals who remained at a lower BMI even years after immigration. In other words, not everyone became obese years after immigration.

As suggested by the reviewer, to test the potential impact of these specific individuals, we performed sensitivity tests by excluding participants with low BMI (<25kg/m^2^) after years of US residence (high US exposure group) and excluding participants with high BMI (>=30 kg/m^2^) in the low US exposure groups. As shown in the Table below, all of the paradoxical patterns remained the same in the sensitivity analyses.

Table. Associations of 23 genera with BMI and US exposure in the sensitivity analyses.

**       Page 8, Line 170: Suggest including a definition of what* *microbial co-occurrence exactly is and what it means in this context. Further, why should studies adjust for microbial co-occurrence? Perhaps include a brief explanation as to why this should happen.*

***Response 26:*** We thank the reviewer for these constructive suggestions. As suggested, we have added more information on microbial interaction and co-occurrence in the Introduction section and added the definition of microbial co-occurrence in the main text (Line113; Line 553-). Microbial co-occurrence network, which infer ecological associations based on taxonomic composition data obtained from high-throughput sequencing techniques (Hirano H, et al., BMC Bioinformatics. 2019), are widely used to visualize the statistically significant associations between microbes in microbial communities and predict the potential microbe interactions (Ma B, et al., Microbiome. 2020). In this study we performed Spearman rho based co-occurrence network analysis with Cytoscape (Otasek D, et al., Genome Biol. 2019), to explore the relationships among the identified bacterial taxa and better describe the overall structure of microbial components. Nodes and edges represent bacterial genera and statistically significant correlations between these genera, respectively.
In addition, we have added a paragraph in the Discussion section to explain why microbe interactions needs to be considered in future studies (Line 385- ).

**       Page 9, Line 193: What results are being referred to here: "we examined the associations of these genera with two other* *obesity measures, body fat percentage and waist circumference, and severity of obesity, and the results were consistent". In other words, what was the consistent result? Suggest defining this so that it is clearer for the reader. Also, how is "severity of obesity" defined or what does it refer to exactly? BMI classification? Is this not already assessed in the initial analysis?*

***Response 27:*** We thank the reviewers for the comments and questions. In our primary analyses, we used BMI as a main obesity measure, then we also examined other two obesity measures, body fat percentage (BFP) and by waist circumference, to further support our results on the associations between gut microbiota and host obesity. As shown in **Figure S6**, the associations of gut microbial general with BMI, BFP and waist circumference were generally consistent results. We have revised this sentence in main text to make it clearer (line 223-).

The “severity of obesity” was defined by BMI: normal weight (18.5 ≤BMI <25 kg/m^2^), overweight (25≤BMI <30 kg/m^2^), obese I (30≤BMI <35 kg/m^2^), obese II (35≤BMI <40 kg/m^2^), and obese III (BMI ≥40 kg/m^2^). We have added the descriptions in the Method section (line 483- ). In our initial analysis, we treated BMI as a continues variable in the model. Here we examined “severity of obesity” based on BMI as a categorical variable. These results have been shown in **Table S12 and described in the main text (line 223-).**

**10       Page 9, Line 199: "…also observed significant positive correlations among Coprococcus, Roseburia, Prevotella and Dorea" Does this mean these genera co-occur? It is unclear what this means.*

***Response 28:*** Yes, the positive correlations among these genera suggest that these genera might co-occur. And the potential co-occurrence among these genera was further demonstrated in the co-occurrence network **(Figure 2C**) in which these genera were clustered. We have revised this sentence in the main text to make it clearer (Line 233-).

**       Page 12, Line 264: In the corresponding figure, it doesn't seem like there was much of a difference among the immigrant groups but only a significant decrease in the US-born group. Can the authors comment on whether there were significant differences among immigrant groups?*

***Response 29:*** We thank the reviewer for this very helpful comment. As suggested, we performed further analyses only among immigrant groups and found that there was a decreasing trend of enzyme K15531 across increased years of US residence, but the significance was marginal (P=0.10) which might be due to reduced power after removing the US-born group in the analyses. This may also suggest the significant association between US exposure and the enzyme K15531might be mainly driven by the US-born group. In the revised manuscript, we have described these new results in the main text (Line 312-).

**       Page 14: Lines 318-324: The authors write here about strains within the species Prevotella, but provide absolutely no analysis of those in this manuscript. It would be good to include a strain analysis if it is discussed. This is especially important as there is a claim here that the "co-occurrence hypothesis" is better than the "multiple strain hypothesis".*

***Response 30:*** we thank the reviewer for the comment and apology the unclear statement, as we did not intend to claim the "co-occurrence hypothesis" is better than the "multiple strain hypothesis". The "co-occurrence hypothesis" might be another explanation for the paradoxical associations in addition to the *"multiple strain hypothesis",* and both hypotheses need further investigation in future studies. In the revised manuscript, we have revised our discussion focusing on previous studies regarding strain variation, as we do not have strain-level data due to the limitation of shallow shotgun metagenomics (Line 410-). Also, we have made it clear that our findings provide another potential explanation for the unexpected associations between *Prevotella* and human health and disease observed in our and other studies (Line 376-).

**       Page 15, Line 327: This last sentence of the paragraph is hard to understand. Are the authors saying that Prevotella does not truly correlate with/influence health but rather it is the taxa that correlate with it instead?*

***Response 31:*** we thank the reviewer for pointing this out and have revised this sentence: (Line 376-). “Our study provides another potential explanation. The inconsistent associations between *Prevotella* and human diseases (e.g., obesity) across different populations, might be due to variation in correlations between *Prevotella* and the true disease-associated taxa, rather than *Prevotella* itself.”

**       Page 17, Line 371: Were participants excluded if they used antibiotics/probiotics, or was this simply recorded in the study? If it is the former, please include this information. If it is the latter, how was this controlled for in the study?*

***Response 32:*** We thank the reviewer for this question. In this study, we ascertained “the use of antibiotics within the prior 6 months” by self-administered questionnaire at the time of stool sample collection. In our primarily analysis, we controlled for antibiotic use by including it as a covariate in the multi-variable linear regression models. This has been described in the main text (Methods section, line 546-).

In the revised manuscript, we further conducted a sensitivity analysis by excluding individuals with antibiotic use. As shown in the Figures below associations of taxa with host obesity and US immigration were highly consistent in all samples with adjustment for antibiotic use and in participants without antibiotic use. We have mentioned these sensitivity analysis results in the main text (Line 180-).

**Figure. Sensitivity analyses excluding individuals with antibiotic use.**

**       Figures (overall): If possible, perhaps the figures with the red and green could be changed to red and blue (e.g. Figure 1A, 2B, 3D, 4B). The red and green might be difficult to differentiate for people with colorblindness.*

***Response 33:*** We thank the reviewer for this considerate suggestion. We have changed the color to red and blue style for these 4 Figures (i.e. **Figure 1A, Figure 2B, Figure 3D, and Figure 4B**) in the revised manuscript.

**       Figure 1A: Can something be added to this figure to show which ring represents BMI and which represents US immigration? It's a bit confusing until you read the figure caption, so if something can be added to the figure this would aid in understanding.*

***Response 34:*** Thanks. We re-draw the **Figure 1A** and added two labels to better indicate the BMI ring and US exposure ring.
 **       Figure 2A: Please include a label for the two clusters and which one refers to BMI and which one refers to US immigration.*
 ***Response 35:*** We would like to clarify that in **Figure 2A** the “Cluster A” were consist of 9 correlated bacteria genera which associated with higher BMI and less US exposure in the multivariable linear regression models, while the “Cluster B” were consist of 8 correlated bacteria genera which associated with lower BMI and greater US exposure.

For each of the individual genus, the associations with BMI and US exposure (multi-variable models) were shown in the 1^st^ and 2^nd^ column of **Figure 2B** ; and the associations with BMI and US exposure (Conditional analyses mutual adjustment model ) were shown in the 3^rd^ and 4^th^ column of **Figure 2B**. In the updated figure, we have used “Triangle” to indicate genera that were independently associated with BMI, and used “Square” to indicate genera that were independently associated with US exposure.

*** *Figure 2B: At first, it wasn't obvious that the taxa listed in Figure 2A also were aligned with what was being depicted in Figure 2B. It would improve understanding if the colours aligned as well (i.e. the red taxa align with the red boxes indicating those taxa associate with BMI; perhaps use dark blue in Figure 2B instead of green).*

***Response 36:*** Thanks. We re-draw the **Figure 2A and 2B** and changed the color to red and blue style. We also re-formatted the name of genera , to highlight that the taxa listed in **Figure 2A** also were aligned with what was being depicted in **Figure 2B**.

**Second round of review**

**Reviewer 2**

For the most part, I am happy with this revised version, and have only minor comments. On page 6, the authors state how obesity and US exposure reaches R2 values of 0.9 and 1.8%. The authors then continue to discuss "other factors", and state that with an R2 value of <2%, their importance is small. The authors should rewrite this to make clear that the importance of all factors, including obesity and US exposure, are small. I still struggle with the term 'clusters', that is used at different positions in the paper (for example page 9 and page 13). Are these really 'clusters', which implies that some sort of clustering approach was used, or are these not just 'groups' (meaning, the taxa or functions that are either positively or negatively associated with variables? Not sure I like the term 'marginally significant'. Perhaps their is a trend.

**Point-by-point Response to the reviewers’ further comments:**

All changes to the manuscript are marked in the text.

***Reviewer #2:***
*For the most part, I am happy with this revised version, and have only minor comments.

On page 6, the authors state how obesity and US exposure reaches R2 values of 0.9 and 1.8%. The authors then continue to discuss "other factors", and state that with an R2 value of <2%, their importance is small. The authors should rewrite this to make clear that the importance of all factors, including obesity and US exposure, are small.*
***Response 1:*** We thank the reviewer for this helpful suggestion. We have rewritten this sentence. Page 7.“…while all these R^2^ values were relatively small (R^2^<2.0%), including those for obesity and US exposure (Additional file 1: Figure S2).”

*I still struggle with the term 'clusters', that is used at different positions in the paper (for example page 9 and page 13). Are these really 'clusters', which implies that some sort of clustering approach was used, or are these not just 'groups' (meaning, the taxa or functions that are either positively or negatively associated with variables?*
***Response 2:*** We thank the reviewer for this question. We performed hierarchical clustering approach to identify the clusters among bacterial genera, and showed cluster dendrogram (e.g. Figure 2A) in addition to the correlation heatmap. We have added the description in Method section (Page 25).

*Not sure I like the term 'marginally significant'. Perhaps their is a trend.*
***Response 3:*** We thank the reviewer for this considerate suggestion. We have rewritten the sentence and deleted this term. Page14. “The inverse association between oligosaccharide reducing-end xylanase and US exposure might be mainly driven by relatively lower levels of oligosaccharide reducing-end xylanase in US-born Hispanics/Latinos compared to immigrant Hispanics/Latinos, as we only found a trend for the inverse association between oligosaccharide reducing-end xylanase and years of US residence among immigrant Hispanics/Latinos (P=0.10).”
